# Effectiveness of robot therapy on body function and structure in people with limited upper limb function: A systematic review and meta-analysis

**DOI:** 10.1371/journal.pone.0200330

**Published:** 2018-07-12

**Authors:** Fernanda Márcia Rodrigues Martins Ferreira, Maria Emília Abreu Chaves, Vinícius Cunha Oliveira, Adriana Maria Valladão Novais Van Petten, Claysson Bruno Santos Vimieiro

**Affiliations:** 1 Programa de Pós-Graduação em Engenharia Mecânica, Bioengineering Laboratory, Universidade Federal de Minas Gerais, Belo Horizonte, Minas Gerais, Brazil; 2 Bioengineering Laboratory, Universidade Federal de Minas Gerais, Belo Horizonte, Minas Gerais, Brazil; 3 Pós-Graduação em Reabilitação e Desempenho Funcional, Universidade Federal dos Vales do Jequitinhonha e Mucuri, Diamantina, Minas Gerais, Brazil; 4 Department of Occupational Therapy, Universidade Federal de Minas Gerais, Belo Horizonte, Minas Gerais, Brazil; 5 Graduate Program in Mechanical Engineering, Universidade Federal de Minas Gerais, Belo Horizonte, Minas Gerais, Brazil; 6 Graduate Program in Mechanical Engineering, Pontifícia Universidade Católica de Minas Gerais, Belo Horizonte, Minas Gerais, Brazil; IRCCS E. Medea, ITALY

## Abstract

Robot-Assisted Therapy (RT) is an innovative approach to neurological rehabilitation that uses intensive, repetitive, interactive, and individualized practice. This systematic review aimed to investigate the effectiveness of RT on the body function and structure of people with upper limb impairments (PROSPERO registration: CRD42017054982). A search strategy conducted on seven databases identified randomized controlled studies. Methodological quality was assessed using the PEDro scale. When possible, the data were pooled, the strength of evidence was assessed using the GRADE system, and the effect sizes were assessed using Cohen coefficient. Subgroup analyses investigated the impact on the estimated effects of the following parameters: methodological quality; portion of the assessed upper limb; duration of stroke; and intervention dose and duration. Thirty-eight studies involving 1174 participants were included. Pooled estimates revealed small effects of RT on motor control and medium effects on strength compared with other intervention (OI) at a short-term follow-up. Standardized differences in means were as follows: 0.3 (95% CI 0.1 to 0.4) and 0.5 (95% CI 0.2 to 0.8). Effects at other time points and on other investigated outcomes related to body function and structure were not found (*p*>0.05). The strength of the current evidence was usually low quality. Subgroup analyses suggested that the methodological quality, and duration and dose of RT may influence the estimated effects. In conclusion, RT has small effects on motor control and medium effects on strength in people with limited upper limb function. Poor methodological quality, and lower treatment dose and duration may impact negatively the estimated effects.

## Introduction

Upper limb motor impairments following a neurological disorder are common and may lead to function limitations, dependence and poor quality of life among the affected people[1]. There are many rehabilitation programs aiming to promote the function, independence and social reintegration of these affected people[2]. These programs include constraint-induced movement therapy, electromyographic biofeedback, mental practice with motor imagery, repetitive task training, functional electrical stimulation and Robot-Assisted Therapy (RT)[3,4,5].

RT is an innovative approach to neurological rehabilitation that involves intensive, repetitive, interactive, and individualized practice[6]. The use of RT for upper limb disorders dates to the 1990s. Since then, a number of robotic devices have become commercially available to clinics and hospitals worldwide[7].

Previous reviews have suggested that RT improves upper limb motor control and muscle strength[8,9,10,11,12]. However, these studies drew limited conclusions about the effectiveness of RT on the body function and structure of people with upper limb impairments. It was not possible to specify comparisons; the use of RT alone or combined with other interventions was compared with minimal or other interventions. Other limitations included few investigated outcomes related to body function and structure in individuals with stroke, absence of protocol registration and assessment of the strength of evidence[11], language restriction for the included studies, and absence of medium- and long-term effects[12].

It also remains unknown whether the estimated effects of RT are impacted by the portion of the assessed upper limb (i.e., proximal shoulder-elbow level or distal hand-wrist level)[9,10], treatment dose and/or duration[11,12], or the methodological quality of the studies. Therefore, the aim of this systematic review was to investigate the effectiveness of RT on outcomes related to body function and structure of people with upper limb impairments at short-, medium- and long-term follow-ups. The potential impacts of the portion of the assessed upper limb, duration of stroke, treatment dose and/or duration, and methodological quality were also investigated.

## Methods

### Search strategy and inclusion criteria

The protocol of this review was prospectively registered at PROSPERO (CRD42017054982). The search for relevant studies was conducted in PEDro (Physiotherapy Evidence Database), EMBASE (Excerpta Medica Database), Medline (Medical Literature Analysis and Retrieval System Online), CINAHL (Cumulative Index to Nursing and Allied Health Literature), Cochrane (Cochrane Collaboration), AMED (Allied and Complementary Medicine Database) and Compendex (Compendex Engineering Index) without language or date restrictions. In addition, a hand search was conducted in reference lists of previous reviews in this area. The search terms were related to “Robot-Assisted Therapy” (robotics, orthotic devices, bionic device, exoskeleton, robotic aided therapy, therapy computer-assisted, robot-assisted, robotics-assisted, self-help devices, robotic device, dynamic orthotic device, robot-mediated therapy, robot-supported, computer-assisted instruction, computer aided, computer-aided design, computer assisted, artificial limb, rehabilitation robotics, human-robot interaction, robot-aided rehabilitation, robotic rehabilitation, orthosis, taping, splinting, assistive technology devices, assistive device therapy), “upper limb” (upper extremity, arm, arm injuries, hand, hand injuries, shoulder, shoulder injuries, elbow, axilla elbow, forearm injuries, forearm, finger, finger injuries, wrist injuries, wrist) and “randomized controlled trial” (random allocation, double blind method, single blind method, placebo, random, controlled clinical trial, clinical trial, comparative study, evaluation study, follow-up study, prospective study, crossover studies). See S1 Appendix in the Addenda for the detailed search strategy.

This review included prospective randomized or quasi-randomized controlled studies including inpatients and outpatients from any primary, secondary or tertiary care setting and community. Studies were eligible if they included participants of both sexes, regardless of age, with limited upper limb function caused by stroke. The intervention of interest was RT, which was defined as the application of any electronic, computerized control system connected to mechanical devices designed to perform human functions. Studies were eligible if RT was compared with minimal intervention or other intervention (OI). We defined minimal intervention as when the control group received no intervention, received sham or placebo intervention, or was on a waiting list. We considered any other active intervention that was not robotic therapy, such as conventional therapy and physical therapy. Studies investigating additional effects of RT were also included. The outcomes of interest in this review were those related to body function and structure, according to the International Classification of Functioning, Disability and Health[13]. We considered body function as the physiological functions of body systems, including psychological function and body structure, i.e., anatomical parts of the body, such as organs, limbs and their components[14].

### Selection of studies

After removing duplicate studies, the relevant retrieved titles and abstracts were selected. Then, we assessed the potential full texts, and studies fulfilling the eligibility criteria were included.

### Methodological quality assessment

We assessed the methodological quality of the included studies using the 0 to 10 PEDro scale, with higher scores indicating greater methodological quality. Disagreements were resolved by consensus. When available, we used scores already on the PEDro database (http://www.pedro.org.au/).

### Data extraction

We extracted data on the following characteristics of the included studies: number of participants; mean age; percentage of female; cause of the upper limb disorder and its duration; evaluated joints; type of RT; comparison groups; frequency and total duration of intervention; and outcome measures.

The included studies investigated many different outcomes related to body function and structure. For the feasibility of this review, we arbitrarily decided to include the following five most investigated outcomes in the literature: motor control; strength; spasticity; range of motion; and pain. When a given study evaluated these outcomes with more than one instrument, we considered the most consistent instrument among the included studies. When a given study investigated two different RT groups[15–27], we considered both groups, consistent with previous reviews in this area[12, 28]. We extracted data for the complete upper limb, and we separately considered the proximal (i.e., elbow and shoulder) and distal (i.e., wrist and hand) portions of the assessed upper limb, as suggested by previous reviews[9,10]. When more than one measurement was available for the proximal and/or distal upper limb, we considered the elbow and wrist due to their greater consistency among the included studies and clinical implications[29].

The following outcome data were included: sample size, mean and standard deviation (SD) for each group were extracted at the short-, medium- and long-term follow-ups: ≤ 3 months after baseline for short-term; > 3 months but < 12 months after baseline for medium-term; and ≥ 12 months after baseline for long-term. When multiple time points were available within the same follow-up period, the time point closer to the end of the intervention was used for short-term follow-up, that closer to 6 months was used for medium-term follow-up and that closer to 12 months was used for long-term follow-up. SDs were not available in certain included studies, and in those cases, the SDs were imputed from the 95% confidence interval (CI), standard error (SE), *p* value, interquartile range and average from other included studies with similar sources of participants. See S2 Appendix in the Addenda for the detailed extracted data.

### Data analysis

Data for each outcome were pooled when there was sufficient homogeneity among studies. Homogeneity among studies was assessed using *I*^*2*^ statistics. Low heterogeneity was defined as if *I*^*2*^ ≤ 50%, and moderate to high heterogeneity was defined as *I*^*2*^ > 50%[30]. Pooled effects were estimated using standardized mean differences (SMDs) with 95% confidence intervals (CI). A fixed-effects model was used to conduct the meta-analysis when *I*^*2*^ ≤ 50%, and a random-effects model was used to conduct the meta-analysis when *I*^*2*^ > 50%. To judge the clinical relevance of RT, the effect size was assessed using Cohen’s d coefficient according to the following parameters:

0.2 as small effect, 0.5 as medium effect, and 0.8 as large effect [31]. A funnel plot was used to investigated publication bias when at least 10 studies were pooled[30]. The meta-analysis was performed using the software Comprehensive Meta-Analysis, version 3.3.070.

The GRADE (Grading of Recommendations Assessment, Development and Evaluation) system was used to summarize the overall quality of evidence for each outcome[32]. We rated evidence from the high-quality level and downgraded it one point if one of the following pre-specified criteria was present: low methodological quality (average PEDro score < 6); inconsistency of estimates among pooled studies (*I*^*2*^ > 50%) or when its assessment was not possible (no pooling); indirectness of participants (over 50% of the studies did not describe inclusion criteria); and imprecision (pooling < 300 participants for each outcome)[33].

Subgroup analyses were used to investigated the impact of the following on estimated effects: 1) poor methodological quality (i.e., removing studies with score of five or less out of ten on PEDro scale); 2) investigated portion (proximal and distal portions of the upper limb); 3) duration of stroke (duration of the current episode ≤ 6 months and > 6 months); and 4) treatment dose and duration. For dose, analyses investigated whether the effects of studies providing the same amount of intervention differed from those providing different amounts of intervention for RT and control groups. For duration, analyses investigated whether the effects of studies providing > 20 sessions differed from those providing ≤ 20 sessions. The impact of dose and duration was also investigated using total volume (i.e., number of sessions x time per session in hours), dichotomized into studies with total volume > 20 hours of intervention and those ones with ≤ 20 hours. We arbitrarily decided on these cut-offs because they were the most frequently used cut-offs in the included studies. Subgroup analyses were conducted to compare RT and OI at the short-term follow-up because this was the most investigated follow-up.

## Results

### Flow of studies through the review

**Fig 1:**
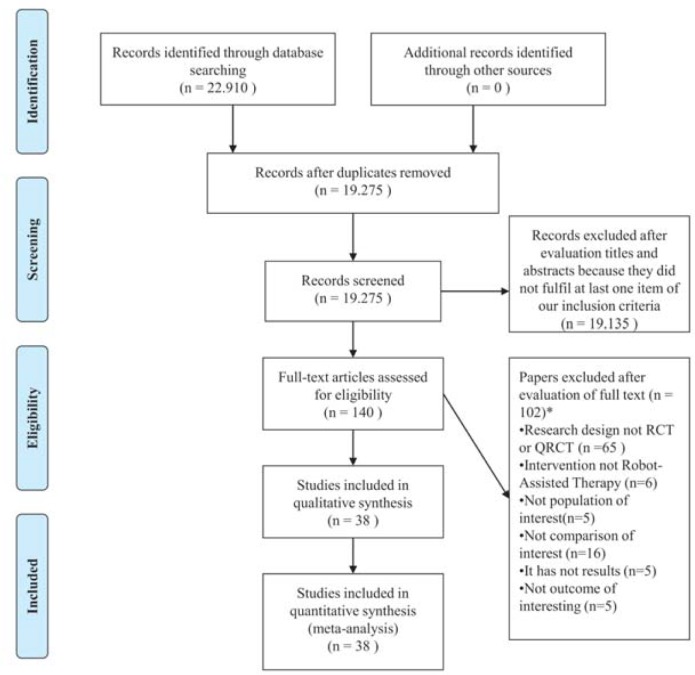
Flow chart of studies through the review. *Papers may have been excluded for failing to meet more than one inclusion criteria. Abbreviations: RCT = randomized controlled trials; QRCT = quasi-randomized controlled trials.

The searches retrieved 22910 references. After removing duplicates, 19275 titles and abstracts were screened. Of these, 19135 were excluded, and 140 potential full texts were assessed. The hand search did not retrieve additional studies. Finally, 38 original studies were included[15–27,34–57]. Fig 1 presents the flow of studies through the review.

### Characteristics of studies

The characteristics of the included studies are presented in Table 1. All included studies were prospective randomized controlled studies published in English between 1997 and 2015. The 38 original studies enrolled 1174 participants of both sexes, with a mean age ranging from 51.2 to 57.8 years. The cause of the upper limb disorder was stroke, with 24 of the 38 studies including people with chronic episodes of this health condition.

**Table 1:** Characteristics of the included studies (n = 38).

Study	Health Condition	Source	Participants	Intervention	Duration and frequency	Outcome measures	Robotic Device
Abdullah et al. (2011)	Individuals with unilateral stroke, aged between 16–90 years, 2 to 8 weeks post stroke.	Recruited at Chedoke Stroke Rehabilitation Unit at Hamilton Health Sciences in Ontario.	n = 19 Age (*yr*) = N/A (SD = N/A) Gender = 8M / 11F	Exp RT = robotic therapy (n = 8) OI = conventional therapy (n = 11)	Exp RT = 45 min/session; 3/wk x 8-11wk OI = 45 min/session; 3/wk x 8–11 wk	Motor control: Chedoke McMaster Stroke Assessment of the arm and hand range 1–7 Pain: Chedoke McMaster Stroke Assessment Pain Inventory Scale range 1–7 Follow-up = post-treatment	Robotic System
Aisen et al. (1997)	Individuals with a single stroke, 3 weeks post stroke.	Recruited at Burke Rehabilitation Hospital in New York.	n = 20 Age (*yr*) = N/A (SD = N/A) Gender = 11M / 9F	Exp RT = robotic therapy + conventional therapy (n = 10) MI = sham robotic therapy + conventional therapy (n = 10)	Exp RT = 60 min/session; 5/wk MI = had weekly to biweekly contact with the robotic device	Motor control: Fugl-Meyer range 0–66 Strength: Motor power shoulder and elbow (in the biceps, triceps, and anterior and lateral deltoid muscles) range 0–20 Follow-up = post-treatment	MIT- MANUS
Ang et al. (2014)	Individuals with stroke for at least 4 months, aged between 21–80 years.	Recruited at Tan Tock Seng Hospital in Singapore.	n = 21 Age (*yr*) = 54.2 (SD = 12.4) Gender = 14M / 7F	Exp RT = robotic therapy (n = 8) OI = standard arm therapy (n = 7)	Exp RT = 90 min/session; 3/wk x 6 wk OI = 90 min/session; 3/wk x 6 wk	Motor control: Fugl-Meyer range 0–66 Follow-up = post-treatment and 6, 8 weeks	Haptic Knob (HK)
Brokaw et al. (2014)	Individuals with stroke for at least 6 months.	Recruited through the MedStar National Rehabilitation Hospital stroke database.	n = 10 Age (*yr*) = 57 (SD = 11.7) Gender = N/A	Exp RT = robotic therapy (n = 7) OI = conventional therapy (n = 5)	Exp RT = 12 h x 4 wk OI = 12 h x 4 wk	Motor control: Fugl-Meyer range 0–66 Follow-up = post-treatment	ARMin III and HandSOME device
Burgar et al. (2000)	Individuals with chronic stroke at least 6 months.	Recruited and the informed consent was obtained in compliance with Veterans Affairs and Stanford University.	n = 21 Age (*yr*) = N/A (SD = N/A) Gender = 14M / 7F	Exp RT = robotic therapy (n = 11) OI = conventional therapy (n = 10)	Exp RT = 60 min/session; 3/wk x 8 wk OI = 60 min/session; 3/wk x 8 wk	Motor control: Fugl-Meyer total range 0–66 shoulder/elbow range 0–42 wrist/hand range 0–24 Follow-up = post-treatment	Mirror Image Movement Enabler (MIME)
Burgar et al. (2011)	Individuals with acute stroke	Recruited through the Veterans Affairs (VA) Medical Center (Texas), the VA Greater Los Angeles Healthcare System (California) and the VA Palo Alto Health Care System (California).	n = 54 Age (*yr*) = N/A (SD = N/A) Gender = N/A	Exp RT = high dose robotic therapy (n = 17) Exp RT = low dose robotic therapy (n = 19) OI = conventional therapy (n = 18)	Exp RT = 1 h/session x 30 sessions; 3 wk Exp RT = 1 h/session x 15 sessions; 3 wk OI = 1 h/session x 15 sessions; 3 wk	Motor control: Fugl-Meyer total range 0–66 shoulder/elbow range 0–42 Strength: Motor Power range 0–70 Spasticity: Modified Ashworth Scale range 0–5 Follow-up = post-treatment and 24 weeks	Mirror Image Movement Enabler (MIME)
Byl et al. (2013)	Individuals with unilateral stroke for at least 6 months, aged between 25–75 years.	Recruited at University of California in San Francisco.	n = 15 Age (*yr*) = N/A (SD = N/A) Gender = 13M / 2F	Exp RT = unilateral robotic therapy (n = 5) Exp RT = bilateral robotic therapy (n = 5) OI = task specific repetitive training (n = 5)	Exp RT = 90 min/session; 2/wk x 6 wk Exp RT = 90 min/session; 2/wk x 6 wk OI = 90 min/session; 2/wk x 6 wk	Motor control: Fugl-Meyer range 0–66 Spasticity: Modified Ashworth Scale range 0–25 Strength: manual muscle testing elbow range 0–5 Pain: Visual Analogue Scale range 0–10 ROM: total passive range of motion, as the sum of shoulder flexion, abduction, internal rotation and external rotation, elbow flexion and extension and wrist extension and flexion. Range 0–810°. Separate passive range of motion elbow flexion 0–140°. Follow-up = post-treatment	UL-EXO7
Conroy et al. (2011)	Adults with chronic stroke	Community-dwelling adults were recruited.	n = 62 Age (*yr*) = 57.8 (SD = 10.7) Gender = N/A	Exp RT = robotic therapy planar (n = 20) Exp = robotic therapy planar with vertical (n = 18) OI = intensive conventional arm exercise (n = 19)	Exp RT = 60 min/session; 3/wk x 6 wk Exp RT = 60 min/session; 3/wk x 6 wk OI = 60 min/session; 3/wk x 6 wk	Motor control: Fugl-Meyer range 0–66 Follow-up = post-treatment and 12 weeks	MIT-MANUS
Daly et al. (2005)	Individuals with stroke for at least 12 months.	Recruited through the Louis Stokes Cleveland Department of Veterans Affairs Medical Center.	n = 12 Age (*yr*) = N/A (SD = N/A) Gender = 9M / 3F	Exp RT = robotic therapy + motor learning (n = 6) OI = functional neuromuscular stimulation + motor learning (n = 6)	Exp RT = robotic therapy (90 min) + motor learning (210 min); 5/wk x 12 wk OI = functional neuromuscular stimulation (90 min) + motor learning (210 min); 5/wk x 12 wk	Motor control: Fugl-Meyer range 0–66 Follow-up = post-treatment and 24 weeks	InMotion2 (Interactive Motion Technologies, Inc, Cambridge, Massachusetts)
De Araújo et al. (2011)	Individuals with a single unilateral stroke for at least 3 months, aged ≥18 years and exhibited hemiparesis of the right side.	Recruited at University of Pernambuco.	n = 12 Age (*yr*) = N/A (SD = N/A) Gender: 10M / 2F	Exp RT = robotic therapy (n = 6) OI = physical therapy (n = 6)	Exp RT = 50 min/session; 3/wk x 8 wk OI = 50 min/session; 3/wk x 8 wk	Motor control: Fugl-Meyer total range 0–66 shoulder/elbow range 0–36 wrist/hand range 0–24 Spasticity: Modified Ashworth Scale elbow range 0–5 and wrist/hand range 0–5 Follow-up = post-treatment	Electromechanical device (Exoskeleton and static orthosis and Glove)
Fasoli et al. (2004)	Individuals with acute stroke, aged between 27–83 years.	Recruited at Burke Rehabilitation Hospital.	n = 56 Age (*yr*) = N/A (SD = N/A) Gender = 30M / 26F	Exp RT = robotic therapy + conventional rehabilitation (n = 30) MI = assisted or assisted active movement (exposure robotic therapy) + conventional rehabilitation (n = 26)	Exp RT = 60 min/session; 5/wk MI = 12 min/session; 5/wk	Motor control: Fugl-Meyer range 0–66 Motor Status Score shoulder/elbow range 0–40 wrist/hand range 0–42 Strength: Medical Research Council Motor Power shoulder flexion and abduction and elbow flexion and extension range 0–20 Follow-up = discharge	MIT-MANUS
Hesse et al. (2005)	Individuals with subacute stroke within the past 4 to 8 weeks.	Recruited from two rehabilitation centers.	n = 44 Age (*yr*) = N/A (SD = N/A) Gender = 20M / 24F	Exp RT = robotic therapy (n = 22) OI = electrical stimulation (n = 22)	Exp RT = 20 min/session; 5/wk x 6 wk OI = 20 min/session; 5/wk x 6 wk	Motor control: Fugl Meyer total range 0–66 shoulder/elbow range 0–42 wrist/hand range 0–24 Strength: Medical Research Council range 0–45 proximal range 0–15 distal range 0–30 Spasticity: Modified Ashworth Scale total range 0–25 proximal range 0–10 distal range 0–15 Follow-up = post-treatment and 18 weeks	Bi-manu-track
Housman et al. (2009)	Adults with a single stroke at least 6 months, with moderate/severe hemiparesis.	Recruited through the RIC Sensory Motor Performance Program in Chicago.	n = 31 Age (*yr*) = N/A (SD = N/A) Gender = 18M / 10F	Exp RT = robotic therapy + occupational therapist (n = 17) OI = conventional therapy + occupational therapist (n = 17)	Exp RT = 60 min/session; 3/wk x 8–9 wk OI = 60 min/session; 3/wk x 8–9 wk	Motor control: Fugl-Meyer range 0–66 ROM: was calculated as the mean distance between a marker placed on the subject’s wrist and 5 targets, following 5 reach attempts to each target. Strength: Grip strength with the Jamar dynamometer range 0–200 Follow-up = post-treatment and 24 weeks	Therapy Wilmington Robotic Exoskeleton (T-WREX)
Hsieh et al. (2011)	Individuals with chronic stroke for at least 6 months.	Recruited from the Departments of Physical Medicine and Rehabilitation of 3 medical centers in Taiwan.	N = 18 Age (*yr*) = N/A (SD = N/A) Gender = 13M / 5F	Exp RT = robotic therapy high intensity (n = 6) Exp = robotic therapy lower intensity (n = 6) OI = conventional therapy (n = 6)	Exp RT = 90–105 min/session; 5/wk x 4 wk Exp RT = 90–105 min/session; 5/wk x 4 wk (half the number of repetitions) OI = 90–105 min/session; 5/wk x 4 wk	Motor control: Fugl-Meyer range 0–66 Strength: Medical Research Council range 0–5 shoulder flexors/abductors, elbow flexors/ extensors, wrist flexors/extensors, and flexors/extensors of the metacarpophalangeal joints, the average MRC score was calculated Follow-up = post-treatment	Bi-manu-track
Kahn et al. (2006)	Individuals with chronic stroke for at least 1 year.	Recruited from outpatient population at the Rehabilitation Institute of Chicago and from a participant database.	n = 19 Age (*yr*) = N/A (SD = N/A) Gender = 11M / 8F	Exp RT add = robotic therapy + conventional therapy (n = 10) OI = Free reaching (n = 9)	Exp RT add = 45 min/session; 3/wk x 8wk OI = 45 min/session; 3/wk x 8wk	Motor control: Chedoke McMaster Stroke Assessment arm section range 1–7 Follow-up = post-treatment and 24 weeks	The Assisted Rehabilitation and Measurement Guide, ARM Guide
Klamroth-Marganska et al. (2014)	Individuals with chronic stroke, for at least 6 months, aged ≥18 years.	Recruited from four clinical centers in Switzerland.	n = 73 Age (*yr*) = N/A (SD = N/A) Gender = 46M / 27F	Exp RT = robotic therapy (n = 38) OI = conventional therapy (n = 35)	Exp RT = 45 min/session; 3/wk x 8wk OI = 45 min/session; 3/wk x 8wk	Motor control: Fugl-Meyer range 0–66 Spasticity: Modified Ashworth Scale range 0–5. Mean values from nine single joint movements: flexion and extension of the elbow, wrist, finger, thumb and flexion of the shoulder. Strength: grip strength with a handheld dynamometer Jamar range 0–200 Follow-up = post-treatment, 16 and 34 weeks	ARMin
Liao et al. (2011)	Individuals with chronic stroke for at least 6 months.	Recruited from Departments of Physical Medicine and Rehabilitation of three medical centers in Taiwan.	n = 20 Age (*yr*) = N/A (SD = N/A) Gender = 13M / 7F	Exp RT add = robotic therapy + training in functional activities + conventional therapy (n = 10) OI = conventional therapy + training in functional activities (n = 10)	Exp RT add = 90–105 min/session; 5/wk x 4wk OI = 90–105 min/session; 5/wk x 4wk	Motor control: Fugl-Meyer range 0–66 Follow-up = post-treatment	Bi-Manu-Track
Lin et al. (2015)	Individuals with chronic stroke, at least 6 months.	Recruited at Taipei Veterans General Hospital in Taiwan.	n = 33 Age (*yr*) = 55.1 (SD = 10.5) Gender = 28M / 5F	Exp RT = robotic therapy (n = 16) OI = conventional therapy (n = 17)	Exp RT = 30min/session; 3/wk x 4wkOI = 30min/session; 3/wk x 4wk	Motor control: Fugl-Meyer total range 0–66 shoulder/elbow range 0–42 wrist/hand range 0–24 Follow-up = post-treatment	Bilateral isometric handgrip force training Ya-May Company
Lo et al. (2010)	Individuals with chronic stroke for at least 6 months, who were 18 years of age or older.	Recruited from four participating Veterans Affairs medical centers.	n = 127 Age (*yr*) = N/A (SD = N/A) Gender = 122M / 5F	Exp RT = robotic therapy (n = 49) OI = usual care different time and frequency (n = 28)	Exp RT = 60 min/session; 3/wk x 12wk OI = 60 min/session; 3/wk x 12wk	Motor control: Fugl-Meyer range 0–66 Spasticity: Modified Ashworth Scale range 0–5 Pain: Visual Analogue Scale range 0–10 Follow-up = 6, 12, 24 and 36 weeks	MIT-MANUS
Lum et al. (2006)	Individuals with a single subacute stroke within the past 1 to 5 months.	Not informed.	n = 30 Age (*yr*) = N/A (SD = N/A) Gender = 20M / 10F	Exp RT = unilateral robotic therapy (n = 9) Exp = robotic therapy bilateral (n = 5) OI = conventional therapy (n = 6)	Exp RT = 60 min/session; 4wk Exp RT = 60 min/session; 4wk OI = 60 min/session; 4wk	Motor control: Fugl-Meyer shoulder/elbow range 0–42 wrist/hand range 0–24 Strength: Motor Power Scale range 0–70 Spasticity: Modified Ashworth Scale proximal range 0–15 and distal range 0–30 Follow-up = post-treatment and 24 weeks	Mirror Image Movement Enabler (MIME)
Masiero et al. (2014)	Individuals with a first, single acute stroke within 15 days, aged ≥18 years.	Recruited from the Stroke Unit in Italy.	n = 30 Age (*yr*) = N/A (SD = N/A) Gender = 20M / 10F	Exp RT add = robotic therapy + conventional therapy (n = 14) OI = conventional therapy (n = 16)	Exp RTadd = 120 min/session; 5/wk x 5wk OI = 120min/session; 5/wk x 5wk	Motor control: Fugl-Meyer total range 0–66 shoulder/elbow range 0–42 wrist/hand range 0–24 Spasticity: Modified Ashworth Scale range 0–5 Strength: Medical Research Council biceps range 0–5 shoulder abduction, elbow flexion, elbow extension, wrist flexion, and extension. Follow-up = post-treatment, 12 and 28 weeks.	NeReBot
McCabe et al. (2015)	Individuals with chronic stroke, for at least 1 year, aged between 21–81 years.	Not informed.	n = 35 Age (*yr*) = N/A (SD = N/A) Gender = 23M / 12F	Exp RT add = robotic therapy + motor learning (n = 12) OI = motor learning (n = 11)	Exp RT add = robotic therapy (90 min) + motor learning (210 min); 5/wk x 12wk OI = 300 min; 5/wk x 12wk	Motor control: Fugl-Meyer total range 0–66 shoulder/elbow range 0–42 wrist/hand range 0–24 Follow-up = post-treatment	In Motion 2 shoulder-elbow Robot
Page et al. (2012)	Individuals with chronic stroke, for at least 12 months, aged between 21–75 years.	Recruited using approved advertisements distributed to local stroke support groups and outpatient rehabilitation clinics.	n = 16 Age (*yr*) = N/A (SD = N/A) Gender = 11M / 5F	Exp RT add = robotic therapy + repetitive task specific practice (n = 8) OI = repetitive task specific practice (n = 8)	Exp RT add = 60 min/session; 3/wk x 8wk OI = 60 min/session; 3/wk x 8wk	Motor control: Fugl-Meyer range 0–66 Follow-up = 1 week post-invertention	Myomo e100
Rabadi et al. (2008)	Individuals with acute stroke, within 4 weeks of admission.	Recruited from a stroke unit in a Burke Rehabilitation Hospital.	n = 30 Age (*yr*) = N/A (SD = N/A) Gender = 19M / 11F	Exp RT add = conventional therapy + robotic therapy (n = 10) OI = occupational therapy (n = 10)	Exp RT add = conventional therapy (180 min) + robotic therapy (40 min); 12 sessions; 5/wk OI = 220 min; 12 sessions; 5/wk	Motor control: Fugl-Meyer shoulder/elbow range 0–42 wrist/hand range 0–24 Strength: Motor power score is obtained by assessing 14 movements at the scapular, shoulder and elbow joints range 0–70 Spasticity: Modified Ashworth Scale across nine groups of arm muscles range 0–45. Pain: Pain Scale of Fugl-Meyer range 0–24 Follow-up = post-treatment	MIT-MANUS
Ramos-Murguialday et al. (2013)	Individuals with chronic stroke, for at least 10 months, aged between 18–80 years.	Recruited from via public information (German stroke associations, rehabilitation centers, hospitals) all over Germany.	n = 30 Age (*yr*) = N/A (SD = N/A) Gender = 18M / 12F	Exp RT = robotic therapy + physiotherapy (n = 16) MI = sham robotic therapy + physiotherapy (n = 14)	Exp RT = 5/wk x 4wk MI = 5/wk x 4wk	Motor control: Fugl-Meyer total range 0–54 shoulder/elbow range 0–30 wrist/hand range 0–24 Spasticity: Modified Ashworth Scale range 0–56 Follow-up = post-treatment	Brain-Machine-Interface arm and hand orthoses ReoGo robotic arm
Reinkensmeyer et al. (2012)	Adults with a single stroke, for at least 3 months.	Recruited through local hospitals and stroke support groups in California.	n = 26 Age (*yr*) = N/A (SD = N/A) Gender = 17M / 9F	Exp RT add = robotic therapy + conventional therapy (n = 13) OI = conventional therapy (n = 13)	Exp RT add = 60 min/session; 3/wk x 8wk OI = 60 min/session; 3/wk x 8wk	Motor control: Fugl-Meyer range 0–66 Strength: Grip strength with a Jamar Hand Dynamometer range 0–200 Follow-up = post-treatment and 12 weeks	Pneu-Wrex
Sale et al. (2014)	Individuals with a first acute stroke, after 30 ± 7 days.	Recruited at San Raffaele Pisana and Auxilium Vitae Rehabilitation Centre in Italy.	n = 53 Age (*yr*) = N/A (SD = N/A) Gender = 31M / 22F	Exp RT = robotic therapy + physiotherapy (n = 26) OI = conventional therapy + physiotherapy (n = 27)	Exp RT = robotic therapy (45 min) + physiotherapy (180 min); 5/wk x 6wk OI = conventional therapy (45 min) + physiotherapy (180 min); 5/wk x 6wk	Motor control: Fugl-Meyer range 0–66 Spasticity: Modified Ashworth Scale elbow range 0–5 ROM: total passive range of motion, as the sum of shoulder and elbow movements (shoulder flexion/extension, abduction, intra/extra rotation and elbow extension) range 0–720 Strength: Motricity Index (MI) as the sum of shoulder and elbow movements (shoulder flexion/extension, abduction, intra/extra rotation and elbow extension) range 0–100. Follow-up = post-treatment	MIT-MANUS
Sale et al. (2014)	Individuals with a first acute stroke, for at least 30 ± 7 days, aged between 18–80 years.	Not informed.	n = 20 Age (*yr*) = N/A (SD = N/A) Gender = 14M / 6F	Exp RT = robotic therapy + physiotherapy (n = 11) OI = conventional therapy + physiotherapy (n = 9)	Exp RT = robotic therapy (40 min) + physiotherapy (180 min); 4/wk x 4/wk OI = conventional therapy (40 min) + physiotherapy (180 min); 4/wk x 4/wk	Motor control: Fugl-Meyer wrist/hand range 0–24 Strength: Medical Research Council hand flexor and extensor muscles range 0–5 Spasticity: Modified Ashworth Scale range 0–5 Follow-up = post-treatment and 12 weeks	Amadeo Robotic System
Simkins et al. (2013)	Individuals with chronic stroke, for at least 6 months, aged between 23–69 years.	Recruited at University of California.	n = 15 Age (*yr*) = N/A (SD = N/A) Gender = N/A	Exp RT = unilateral robotic therapy (n = 5) Exp = robotic therapy bilateral (n = 5) OI = repetitive task practice (n = 5)	Exp RT = 90 min/session; 2/wk x 12wk Exp RT = 90 min/session; 2/wk x 12wk OI = 90 min/session; 2/wk x 12wk	Motor control: Fugl-Meyer range 0–66 ROM: elbow flexion 0–140° wrist flexion range 0–80° Strength: Manual muscle test elbow and wrist range 0–5 Pain: Visual Analogue Scale range 0–10 Follow-up = post-treatment	EXO-UL7
Susanto et al. (2015)	Individuals with chronic stroke, within 6 to 24 months.	Not informed.	n = 19 Age (*yr*) = N/A (SD = N/A) Gender = 14M / 5F	Exp RT = robotic therapy + conventional therapy (n = 9) MI = non-assisted robot + conventional therapy (n = 10)	Exp RT = 60 min/session; 4/wk x 5wk MI = 60 min/session; 4/wk x 5wk	Motor control: Fugl-Meyer total range 0–66 shoulder/elbow range 0–36 wrist/hand range 0–24 Follow-up = post-treatment and 24 weeks	The modified hand exoskeleton robot
Timmermans et al. (2014)	Individuals with chronic stroke, post-stroke time ≥ 8 months, aged between 18–85 years.	Recruited from Adelante Rehabilitation Centre (Hoensbroek, NL).	n = 22 Age (*yr*) = N/A (SD = N/A) Gender = 16M / 6F	Exp RT add = robotic therapy + task-oriented training method (n = 11) OI = arm-hand training program (n = 11)	Exp RT add = 30 min/session; 4/wk x 8wk OI = 30 min/session; 4/wk x 8wk	Motor control: Fugl Meyer range 0–66 Follow-up = post-treatment and 24 weeks	Haptic Master
Volpe et al. (1999)	Individuals with acute stroke.	Recruited from neurologic rehabilitation service.	n = 12 Age (*yr*) = N/A (SD = N/A) Gender = 7M / 5F	Exp RT = robotic therapy + conventional therapy (n = 6) MI = sham robotic therapy + conventional therapy (n = 6)	Exp RT = 60 min/session; 5/wk MI = 60 min/session; 5/wk	Motor control: Fugl-Meyer shoulder/elbow range 0–42 wrist/hand range 0–24 Strength: Motor power scale shoulder/elbow (biceps, triceps, and anterior and lateral deltoid muscles) range 0–20. Follow-up = 144 weeks after discharge	MIT-MANUS
Volpe et al. (2008)	Individuals with chronic stroke and who had impaired arm and hand mobility for at least 6 months.	Recruited form outpatient clinic.	n = 21 Age (*yr*) = N/A (SD = N/A) Gender = 15M / 6F	Exp RT = robotic therapy (n = 11) OI = conventional therapy (n = 10)	Exp RT = 60 min/session; 3/wk x 6wk OI = 60 min/session; 3/wk x 6wk	Motor control: Fugl-Meyer shoulder/elbow range 0–42 wrist/hand range 0–24 Strength: Motor Power Scale shoulder and elbow range 0–70 Spasticity: Modified Ashworth Scale passive movements, across 9 muscle groups range 0–5 Pain: Pain scale from the Fugl-Meyer range 0–24 Follow-up = post-treatment and 12 weeks	MIT-MANUS
Wu et al. (2012)	Individuals with unilateral chronic stroke, for at least 6 months.	Not informed.	n = 42 Age (*yr*) = 54.4 (SD = 9.69) Gender = 32M / 10F	Exp RT = robot-assisted bilateral arm training OI = conventional therapy (n = 14)	Exp RT = 105 min/session; 5/wk x 4wk OI = 105 min/session; 5/wk x 4wk	Motor control: Fugl-Meyer range 0–66 Follow-up = post-treatment	Bi-manu-track
Xu et al. (2012)	Individuals with 6 months to 2 years after a single mild to moderate stroke, aged 55 years and above.	Recruited from outpatients from Zhongda Hospital (affiliated with Southeast University) and Nanjing Tongren Hospital.	n = 18 Age (*yr*) = N/A (SD = N/A) Gender = 11M / 7F	Exp RT = robotic therapy (n = 9) OI = conventional therapy (n = 9)	Exp RT = 3/wk x 16wk OI = 3/wk x 16wk	Strength: Maximum resistive force with WAM control program Follow-up = post-treatment	Barrett WAMTM Arm
Xu et al. (2014)	Individuals with chronic stroke, aged 50 years and over.	Recruited from Zhongda Hospital affiliated Southeast University and Nanjing Tongren Hospital.	n = 45 Age (*yr*) = N/A (SD = N/A) Gender = 27M / 18F	Exp RT = robotic therapy (n = 23) OI = conventional therapy (n = 22)	Exp RT = 120 min/session; 6/wk x 20wk OI = 120 min/session; 6/wk x 20wk	ROM: Passive range of motion with the assistance of WAM or therapist for elbow. Strength: Maximum resistive force Follow-up = post-treatment	Barrett WAMTM manipulator
Yang et al. (2012)	Individuals with unilateral chronic stroke, within 6 months to 5 years, with an average age of 51.29 years.	Not informed.	n = 21 Age (*yr*) = 51.2 (SD = N/A) Gender = 14M / 7F	Exp RT add = unilateral robotic therapy + functional task practice (n = 7) Exp RT add = bilateral robotic therapy + functional task practice (n = 7) OI = standard rehabilitation (90–105 min/session) (n = 7)	Exp RT add = unilateral robotic therapy (75–180 min) + functional task practice (15–20 min); 5/wk x 4wk Exp RT add = bilateral robotic therapy (75–180 min) + functional task practice (15-20min); 5/wk x 4wk OI = standard rehabilitation (90–105 min/session); 5/wk x 4wk	Motor control: Fugl-Meyer total range 0–66 shoulder/elbow range 0–42 wrist/hand range 0–24 Strength: Medical Research Councilproximal (shoulder flexors, abductors, elbow flexors and extensors and distal (flexors and extensors of wrist and fingers) range 0–5. Spasticity: Modified Ashworth Scale range 0–4 Follow-up = post-treatment	Bi-manu-track
Yoo et al. (2013)	Individuals with chronic stroke who had no visual or cognitive problems.	Not informed.	n = 22 Age (*yr*) = N/A (SD = N/A) Gender = 13M / 9F	Exp RT add = robotic therapy + conventional therapy (n = 11) OI = conventional therapy (n = 11)	Exp RT add = robotic therapy (30 min) + conventional therapy (60 min); 3/wk x 6wk OI = conventional therapy (60 min/session); 3/wk x 6wk	Strength: Medical Research Council range 0–5 Follow-up = post-treatment	ReogoTM

n = sample size; SD = standard deviation; Exp = experimental group; Con = control group; N/A = not available; OI = other intervention; MI = minimal intervention; RTP = repetitive task practice; RT = Robot assisted therapy; wk = week(s); yr = year(s); min = minutes; h = hours; ROM = range of motion.

The duration of RT ranged from 2[25] to 20[57] weeks, and the frequency per week varied from 2[16,18] to 6[57] days. The time spent per session of intervention ranged from 0.2[40] to 2[57] hours. The total volume of intervention per week (i.e., number of sessions per week x duration of each session) ranged from 1[54] to 12[57] hours. The total number of sessions ranged from 12[16,25,45] to 120[57], with most studies ranging from 20 to 24. The total duration of the intervention ranged from 6[45] to 240[57] hours. On average, RT sessions occurred three times per week with a total duration of treatment of 8 weeks. For some studies, primarily those comparing RT to minimal intervention, detailed information on the dose and duration of the intervention was not available[35,36,48,54,56].

Thirty-five studies evaluated motor control using three different instruments. Of these 35 studies, 33 (94.2%)[15,16,18–27,35–38,40–41,44–59] used the Fugl Meyer (FM), two studies (5.7%)[34,42] used the Chedoke McMaster Stroke Assessment (CMSA). Fourteen studies[15–17,20,25,26,38,40,43,46,48,50,51,55] evaluated spasticity using the Modified Ashworth Scale (MAS). Twenty-one studies evaluated strength using six different instruments. Of these 21 studies, seven (33.3%)[15,21,39,40,46,51,58] used the Medical Research Council (MRC), three (14.2%)[41,43,49] used the hand-held dynamometer, six (28.5%)[17,20,25,35,54,55] used the Motor Power Scale (MP), two (9.5%) used the Manual Muscle Testing (MMT)[16,18] and the WAM control program[56,57], and one study[51] used the Motricity Index (MI). To homogenize the data, Newton and kilogram-force values were converted into pounds. We arbitrarily decided to use pounds because this was the most consistent unit among the studies. Five studies evaluated range of motion using three different measures. Of these 5 studies, three studies (60.0%)[16,18,50] assessed range of motion using goniometer, one study[57] used the WAM control program, and one study[41] used the mean distance between a marker placed on the participant’s wrist and five targets. Six studies evaluated pain using three different instruments. Of these 6 studies, three studies (50%)[16,18,26] used the Visual Analogue Scale (VAS), two studies (33.3%)[25,56] used the Pain Scale of Fugl-Meyer, and one study[34] used the Chedoke McMaster Stroke Assessment Pain Inventory Scale.

### Methodological quality of the included studies

The detailed methodological quality of the included studies is presented in S1 Table on the Addenda. The mean methodological quality of the 38 studies was 6.0 on the 0 to 10 PEDro scale. Most studies included the following: between-group comparisons (n = 37 studies, 97.3%); precision and variability estimates (n = 35 studies, 92%); group similarity at baseline and assessor blinding (n = 32 studies, 84.1%); and outcome measures for at least 85% of participants (n = 27 studies, 71%). Concealed allocation was presented in 12 studies (31.5%), and intention-to-treat analysis was presented in 10 studies (26.3%). The primary methodological quality issues were related to the blinding of participants and therapists, which was included in only three (7.9%) and two (5.2%) studies, respectively.

### Effects of robot-assisted therapy

Five studies compared RT with minimal intervention (i.e., sham RT[35,48,54], exposure RT[39,52]), twenty three studies compared RT with OI, i.e., conventional therapy[17,19,20,21,24,34,36,37,41,43,45,50,51,55–57], usual care[26], repetitive task practice[16,18], intensive conventional arm exercise program[22], physical therapy[38], electrical stimulation[23,40], and ten studies investigated the additional effects of RT over OI, i.e., RT added to conventional therapy[25,42,44,49,58], standard therapy[46], motor learning[27], repetitive task-specific practice[47], an arm-hand training program[53] and functional task practice[15]. All 38 included studies reported short-term effects, 15 studies (39.4%) reported medium-term effects[17,20,22,23,26,40–43,46,49,51–53,55] and one study[54] reported long-term effects.

#### Robot-assisted therapy versus minimal intervention

**Fig 2:**
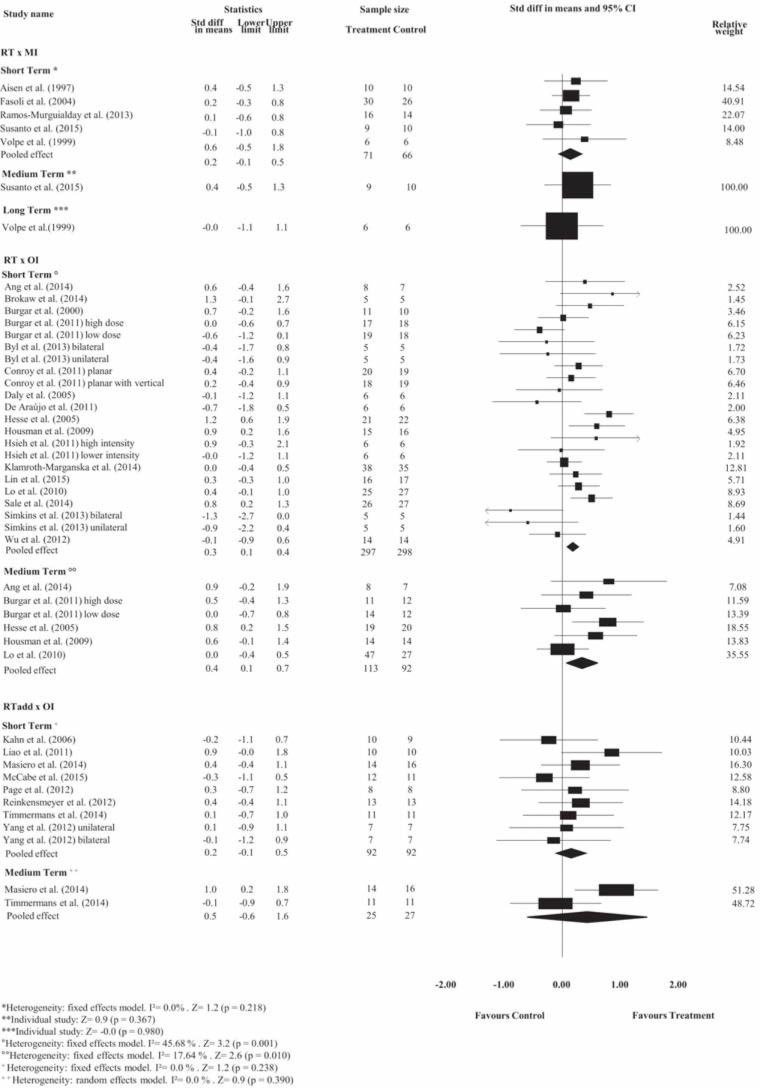
Standardized mean difference (95% CI) comparing RT or additional effect of RT versus MI or OI for motor control of people with limited upper limb function. RT = Robot-assisted therapy; RT add = additional effect of Robot-assisted therapy; OI = other intervention; MI = minimal intervention.

**Fig 3:**
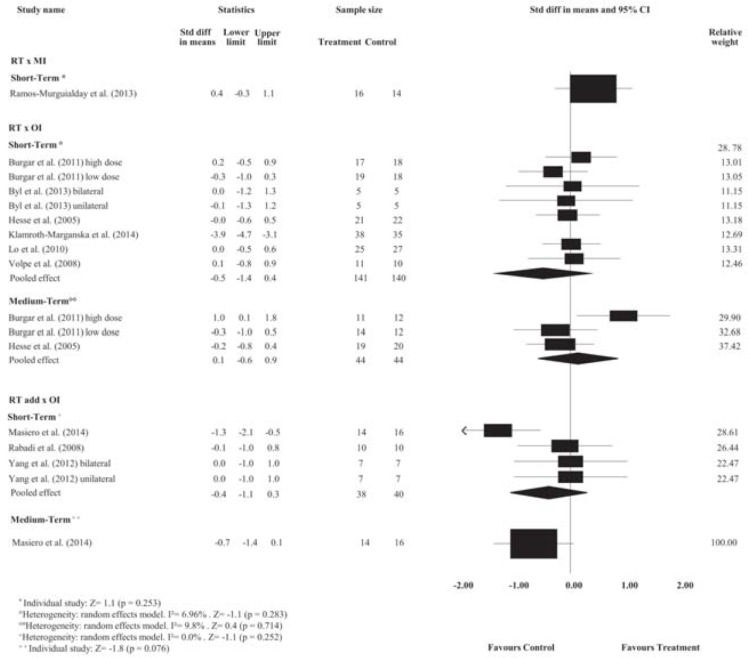
Standardized mean difference (95% CI) comparing RT or additional effect of RT versus OI or MI for spasticity of people with limited upper limb function. RT = Robot-assisted therapy; RT add = additional effect of Robot-assisted therapy; OI = other intervention; MI = minimal intervention.

Pooled estimates showed no effects of RT on motor control at short-, medium- or long-term follow-ups and no effects on spasticity at short-, medium- or long-term follow-ups compared with minimal intervention (*p*> 0.05). Detailed analyses are presented in Figs 2 and 3. The strength of the evidence ranged from low- to very low-quality.

#### Robot-assisted therapy versus other intervention

**Fig 4:**
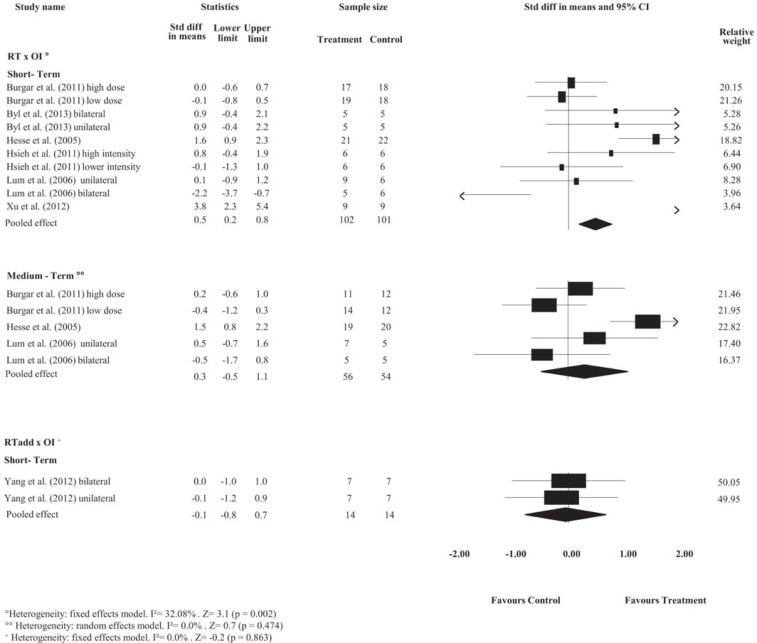
Standardized mean difference (95% CI) comparing RT versus OI for strength in people with limited upper limb function. RT = Robot-assisted therapy; OI = other intervention.

The pooled estimates showed small effects of RT on motor control and medium effects on strength compared with OI at the short-term follow-up. The SMDs were, respectively, 0.3 (95% CI 0.1 to 0.4); and 0.5 (95% CI 0.2 to 0.8). Detailed analyses are presented in Figs 2 and 4. There is high- and very low-quality evidence showing that RT has effects on motor control and strength, respectively, compared to OI at the short-term follow-up.

Pooled estimates showed no effects of RT on spasticity, range of motion and pain at short-term follow-up, or on motor control, spasticity and strength at medium-term follow-up, when compared with OI (*p*> 0.05). Detailed analyses are presented in Figs 2 to 4 and S1 and S2 Figs. The strength of the evidence ranged from low- to very low-quality.

#### Additional effects of robot-assisted therapy over other interventions

Pooled estimates showed no additional effects of RT on motor control, spasticity and pain at short-term follow-up, or on motor control and spasticity at the medium-term follow-up in stroke compared with stand-alone OI (*p*> 0.05). Detailed analyses are presented in Figs 2 and 3 and S1 and S2 Figs. The strength of the evidence ranged from low- to very low-quality.

### Subgroup analysis

We investigated the impact of methodological quality, portions of the assessed upper limb, duration of stroke, and treatment dose and duration on the estimated short-term effects of RT compared with OI (see detailed subgroup analyses in S3 Fig). Methodological quality, and dose impacted the estimated effects for motor control. Poor methodological quality, and lower treatment dose and duration may impact negatively the estimated effects.

## Discussion

This review included 38 studies comparing the efficacy of RT with minimal intervention or OI, and investigating additional effects of RT combined with OI on body function and structure in people with upper limb limitations caused by stroke. RT has small effects on motor control and medium effects on muscle strength. Moreover, the methodological quality, portion of the upper limb, treatment dose, duration and volume may impact the estimated effects. The current low-quality evidence suggests that estimated effects are likely to change with future high-quality studies, and effects are not consistent among outcomes related to body function and structure.

Our findings revealed that compared with OI, RT has statistically significant but small effects on motor control and medium effects on strength. These short-term findings comparing RT with OI are consistent with other reviews on stroke. Veerbeek et al.[12] showed a small improvement on motor control and muscle strength and no effect on spasticity. Prange et al.[8] also found improvement on motor control at the short-term follow-up compared to conventional rehabilitation.

Previous reviews[8,10] did not investigate the medium- and long-term effects. Despite this, Norouzi-Gheidari et al.[10] suggested no effects on motor control at medium-term follow-up when the same doses of RT and OI were used. Our results were consistent with those of the previous study and suggest that upper limb motor control improvement occurs within the short-term (≤ 3 months after stroke)[59]. Moreover, Prange et al.[8] found long-term effects on motor control when RT was used compared to OI. Their findings were not consistent with our results, and a possible explanation is that the previous review[8] included poor-quality studies (i.e., non-randomized controlled studies). As suggested by Norouzi-Gheidari et al.[10], future high-quality studies should confirm our findings because current evidence for the estimated effect is very low and likely to change.

When comparing the efficacy of RT with minimal intervention at different time points, despite trends favouring treatment, the current low-quality evidence showed no significant effects. Susanto et al.[52] stated that there is an insignificant effect, but there are few studies, and they have small samples.

The average methodological quality of the 39 included studies was 6 points on the 0 to 10 PEDro scale, ranging from 2 to 8 points. This quality was consistent with that reported by Veerbeek et al.[12], with an average quality of 6.0 points. The primary methodological issues were related to blinding, which is expected due to the difficulty of fulfilling these criteria in the area of RT.

A subgroup analysis showed that the portions of the assessed upper limb influence the estimated effects only for range of motion. These findings were not consistent with other studies[9,10,12] and were similar to those reported by Mehrholz et al.[11]. Therefore, there is no consensus on the impact of portions of the assessed upper limb, and current evidence is low but likely to change with further high-quality studies that include larger samples. Subgroup analysis also suggested a greater effect on motor control in chronic stroke, similar to most recent review[28]. Subgroup analysis also suggested that when conventional therapy (CT) is used at the same dose as robot-assisted therapy (RT), there is a significant effect on motor control, unlike the findings reported by Kwakell[9]. Subgroup analysis also suggested an impact of the number of sessions and treatment volume on some estimated effects. Greater number of sessions seems to impact motor control, and greater treatment volume seems to impact motor control. The effect of greater treatment dose was suggested by Lohse et al.[60]; however, time as a dose representation is a rather crude estimate and provides no evidence of the actual amount of movement or types of movement, nor does this representation take into account periods of inactivity or rest[61]. In this regard, a previous review indicated that although there is no consensus, the minimum dose should be at least 16 hours of training[62].

This review and the current literature have some potential limitations. First, there is only a small number of randomized controlled studies that mainly investigate range of motion and pain, and few studies comparing RT with minimal intervention at different time points. Second, studies typically had small sample sizes. Third, subgroup analyses did not investigate impact of types of RT devices, and dichotomization was a potential limitation to get full information regarding the impact of the investigated factors.

Further high-quality randomized controlled studies with larger sample sizes are warranted to elucidate more precise effects of RT on outcomes related to body function and structure, especially the long-term effects. Studies comparing RT with minimal intervention should be conducted, since the current evidence is very low-quality. These studies should report the treatment dose and duration. Future studies should investigate whether RT is effective on psychological factors, and other outcomes related to the activity and participation domains in the ICF. In addition, it is imperative to conduct studies on the cost-effectiveness of RT.

In conclusion, RT has small effects on motor control and medium effects on strength in people with limited upper limb function caused by stroke. Poor methodological quality, and lower treatment dose and duration may impact negatively the estimated effects. Clinicians consider this approach because it has few or no side effects. In addition, there may be long-term financial benefits to employing therapeutic robots. The current low-quality evidence suggests that the estimated effects are likely to change with future high-quality studies and that the effects are not consistent among outcomes related to body function and structure.

## Supporting information

S1 Checklist PRISMA checklist. (DOC)

S1 Fig Standardized mean difference (95% CI) comparing RT alone versus OI for range of motion in people with limited upper limb function. RT = Robot-assisted therapy; OI = other intervention.
(EPS)

S2 Fig Standardized mean difference (95% CI) comparing RT or additional effect RT versus OI for pain of people with limited upper limb function. RT = Robot-assisted therapy; RT add = additional effect of Robot-assisted therapy; OI = other intervention.
(EPS)

S3 Fig Subgroup analysis investigating the impact of methodological quality, proximal and distal portions of the upper limb, treatment dose, number of sessions, total volume and duration of stroke on estimated effects of RT versus OI at short-term follow-up. RT = Robot-assisted therapy; OI = other intervention; ROM = range of motion.
(EPS)

S4 Fig Funnel plot of RT versus OI for short-term motor control. (EPS)

S1 Table Methodological quality of the included studies using the PEDro scale. Y = yes; N = no.
(DOC)

S1 Appendix Full search strategy conducted on October 16^th^ 2015. (DOC)

S2 Appendix Extracted data. (DOCX)
