# Correction: Effectiveness of robot therapy on body function and structure in people with limited upper limb function: A systematic review and meta-analysis

**DOI:** 10.1371/journal.pone.0207962

**Published:** 2018-11-16

**Authors:** The PLOS ONE Staff

The Funding section is incorrect. The correct Funding section is: The authors would like to acknowledge the financial support of: FINEP (Financiadora de Estudos e Projetos—Project 01.12.0476.01), CNPq (Conselho Nacional de Desenvolvimento Científico e Tecnológico—Project 550236) and PRPq-UFMG (Pro-Reitoria de Pesquisa da UFMG). The publisher apologizes for the error.
