# Supplementary material for: Effectiveness of robot therapy on body function and structure in people with limited upper limb function: A systematic review and meta-analysis
Source: PLoS One. 2018 Jul 12;13(7):e0200330. doi: 10.1371/journal.pone.0200330 (PMC6042733; doi:10.1371/journal.pone.0200330)
Supplement: S1 Table — Y = yes; N = no. (DOC) [file pone.0200330.s006.doc]

**S1 Table.** Methodological quality of the included studies using PEDro scale.

| Study | Random allocation | Concealed allocation | Groups similar at baseline | Participant blinding | Therapist blinding | Assessor blinding | <15% dropouts | Intention-to-treat analysis | Between-group difference reported | Point estimate and variability reported | Total  (0 to 10) |
| --- | --- | --- | --- | --- | --- | --- | --- | --- | --- | --- | --- |
| Abdullah et al (2011) | Y | Y | Y | N | N | Y | Y | N | Y | N | 6 |
| Aisen et al (1997) | Y | N | Y | Y | Y | N | Y | N | Y | Y | 7 |
| Ang et al (2014) | Y | N | Y | N | N | Y | Y | N | Y | Y | 6 |
| Brokaw et al (2014) | Y | N | N | N | N | Y | N | N | Y | Y | 4 |
| Burgar et al (2000) | Y | N | Y | N | N | Y | Y | N | Y | Y | 6 |
| Burgar et al (2011) | Y | N | Y | N | N | Y | Y | N | Y | Y | 6 |
| Byl et al (2013) | Y | N | Y | N | N | Y | N | N | Y | Y | 5 |
| Conroy et al (2011) | Y | N | Y | N | N | Y | Y | N | Y | Y | 6 |
| Daly et al (2005) | Y | N | Y | N | N | Y | Y | N | N | Y | 5 |
| Araújo et al (2011) | Y | N | Y | N | N | N | Y | N | Y | Y | 5 |
| Fasoli et al (2004) | Y | N | Y | N | N | Y | Y | N | Y | Y | 6 |
| Hesse et al (2005) | Y | Y | N | N | N | Y | Y | Y | Y | Y | 7 |
| Housman et al (2009) | Y | N | Y | N | N | Y | N | N | Y | Y | 5 |
| Hsieh et al (2011) | Y | Y | Y | N | N | Y | Y | Y | Y | Y | 8 |
| Kahn et al (2006) | Y | N | N | N | N | Y | N | N | Y | Y | 4 |
| Klamroth-Marganska et al (2014) | Y | Y | Y | N | N | Y | Y | Y | Y | Y | 8 |
| Liao et al (2011) | Y | Y | Y | N | N | Y | Y | N | Y | Y | 7 |
| Lin et al (2015) | Y | Y | Y | N | N | Y | Y | N | Y | Y | 7 |
| Lo et al (2010) | Y | N | Y | N | N | Y | Y | Y | Y | Y | 7 |
| Lum et al (2006) | Y | N | N | N | N | Y | N | N | Y | Y | 4 |
| Masiero et al (2014) | Y | N | Y | N | N | Y | N | Y | Y | Y | 6 |
| McCabe et al (2015) | Y | N | Y | N | N | Y | Y | Y | Y | Y | 7 |
| Page et al. (2012) | Y | N | Y | N | N | Y | N | N | Y | Y | 5 |
| Rabadi et al (2018) | Y | Y | N | N | N | Y | Y | N | Y | Y | 6 |
| Ramos-Murguialday et al (2013) | Y | Y | Y | Y | Y | N | Y | N | Y | Y | 8 |
| Reinkensmeyer et al (2012) | Y | N | Y | N | N | Y | Y | N | Y | Y | 6 |
| Sale et al (2014) | Y | Y | Y | N | N | Y | Y | Y | Y | Y | 8 |
| Sale et al (2014) | Y | N | N | N | N | Y | Y | Y | Y | Y | 6 |
| Simkins et al (2013) | Y | N | Y | N | N | N | Y | N | Y | Y | 5 |
| Susanto et al (2015) | Y | N | Y | N | N | Y | Y | Y | Y | Y | 7 |
| Timmermans et al  (2014) | Y | Y | Y | N | N | Y | Y | Y | Y | Y | 8 |
| Volpe et al (1999) | Y | N | Y | N | N | Y | N | N | Y | Y | 5 |
| Volpe et al (2008) | Y | N | Y | N | N | Y | Y | N | Y | Y | 6 |
| Wu et al (2012) | Y | Y | Y | Y | N | Y | Y | N | Y | Y | 8 |
| Xu et al (2012) | Y | N | Y | N | N | N | N | N | Y | N | 3 |
| Xu et al (2014) | Y | Y | Y | N | N | N | N | N | Y | N | 4 |
| Yang et al (2012) | Y | N | Y | N | N | Y | Y | N | Y | Y | 6 |
| Yoo et al (2013) | Y | N | Y | N | N | Y | N | N | Y | Y | 5 |

Y = yes; N = no
