# Supplementary material for: Effectiveness of robot therapy on body function and structure in people with limited upper limb function: A systematic review and meta-analysis
Source: PLoS One. 2018 Jul 12;13(7):e0200330. doi: 10.1371/journal.pone.0200330 (PMC6042733; doi:10.1371/journal.pone.0200330)
Supplement: S2 Appendix — (DOCX) [file pone.0200330.s008.docx]

**S2 Appendix** Extracted data

**MOTOR CONTROL (n = 35)**

**Upper limb total**

| **Study** | | **Exercise** | | | | | | ***Comparison** | | | | | | |
| --- | --- | --- | --- | --- | --- | --- | --- | --- | --- | --- | --- | --- | --- | --- |
|  | | **Mean** | | **SD** | | **Sample** | | **Mean** | | **SD** | | | **Sample** | |
| **(exercise vs minimal intervention)** | | | | | | | | | | | | | | |
| ***Short- term*** | | | | | | | | | | | | | | |
| Aisen et al. (1997) | 14.1 | | 9.7 | | | 10 | | 10.1 | | | 11.63 | | | 10 |
| Fasoli et al. (2004) § | 16.8 | | 6.57 | | | 30 | | 15.2 | | | 6.63 | | | 26 |
| Ramos-Murguialday et al. (2013) | 14.56 | | 7.81 | | | 16 | | 13.64 | | | 10.88 | | | 14 |
| Susanto et al. (2015) | 5.11 | | 6.55 | | | 9 | | 5.7 | | | 4.35 | | | 10 |
| Volpe et al. (1999) § | 15.2 | | 10.04 | | | 6 | | 8.6 | | | 11.51 | | | 6 |
| ***Medium- term*** |  | |  | | |  | |  | | |  | | |  |
| Susanto et al. (2015) | 6.11 | | 10.9 | | | 9 | | 2.7 | | | 4.42 | | | 10 |
| ***Long- term*** |  | |  | | |  | |  | | |  | | |  |
| Volpe et al. (1999) § | 20.2 | | 20.33 | | | 6 | | 20.5 | | | 22.05 | | | 6 |
| **(exercise vs other intervention)** | | | | | | | | | | | | | | |
| ***Short- term*** | | | | | | | | | | | | | | |
| Ang et al. (2014) | | 6.5 | | 4.4 | 8 | | 3.6 | | 5.5 | | | 7 | | |
| Brokaw et al. (2014) § | | 27.57 | | 8.77 | 5 | | 18.6 | | 4.22 | | | 5 | | |
| Burgar et al. (2000) § | | 5 | | 4 | 11 | | 2.6 | | 2.4 | | | 10 | | |
| Burgar et al. (2011) high dose § | | 14.4 | | 14.84 | 17 | | 14 | | 15.27 | | | 18 | | |
| Burgar et al. (2011) low dose § | | 6.8 | | 8.28 | 19 | | 14 | | 15.27 | | | 18 | | |
| Byl et al. (2013) bilateral | | 28.2 | | 4.6 | 5 | | 30.6 | | 6.92 | | | 5 | | |
| Byl et al. (2013) unilateral | | 27.8 | | 7.92 | 5 | | 30.6 | | 6.92 | | | 5 | | |
| Conroy et al. (2011) planar§ | | 3.3 | | 3.58 | 20 | | 1.82 | | 3.4 | | | 19 | | |
| Conroy et al. (2011) planar with vertical § | | 2.61 | | 3.44 | 18 | | 1.82 | | 3.4 | | | 19 | | |
| Daly et al. (2005) ¢ | | 32 | | 6.38 | 6 | | 32.5 | | 7.06 | | | 6 | | |
| De Araújo et al. (2011) | | 40.83 | | 6.15 | 6 | | 47.83 | | 13.78 | | | 6 | | |
| Hesse et al. (2005) | | 24.6 | | 14.9 | 21 | | 10.4 | | 7.5 | | | 22 | | |
| Housman et al. (2009) | | 24.9 | | 5.9 | 15 | | 20.03 | | 5 | | | 16 | | |
| Hsieh et al. (2011) high intensity | | 49.33 | | 8.34 | 6 | | 40.33 | | 11.86 | | | 6 | | |
| Hsieh et al. (2011) lower intensity | | 40 | | 10.47 | 6 | | 40.33 | | 11.86 | | | 6 | | |
| Klamroth-Marganska et al. (2014) ‡ | | 23.65 | | 7.1 | 38 | | 23.27 | | 8.2 | | | 35 | | |
| Lin et al. (2015) | | 46.75 | | 16.04 | 16 | | 40.41 | | 19.9 | | | 17 | | |
| Lo et al. (2010) § | | 1.11 | | 5.05 | 25 | | -1.06 | | 5.2 | | | 27 | | |
| Sale et al. (2014) | | 35.46 | | 12.24 | 26 | | 23.96 | | 17.51 | | | 27 | | |
| Simkins et al. (2013) unilateral þ | | 18 | | 4.58 | 5 | | 22 | | 4.36 | | | 5 | | |
| Simkins et al. (2013) bilateral þ | | 16 | | 4.58 | 5 | | 22 | | 4.36 | | | 5 | | |
| Wu et al. (2012) | | 47.14 | | 10.97 | 14 | | 48.57 | | 12.32 | | | 14 | | |
| ***Medium- term*** | |  | |  |  | |  | |  | | |  | | |
| Ang et al. (2014) | | 8.3 | | 5 | 8 | | 3.6 | | 5.9 | | | 7 | | |
| Burgar et al. (2011) high dose § | | 23.6 | | 19.24 | 11 | | 15.3 | | 16.97 | | | 12 | | |
| Burgar et al. (2011) low dose § | | 15.9 | | 13.10 | 14 | | 15.3 | | 16.97 | | | 12 | | |
| Hesse et al. (2005) | | 30 | | 16.8 | 19 | | 16.6 | | 14.9 | | | 20 | | |
| Housman et al. (2009) | | 3.6 | | 3.9 | 14 | | 1.5 | | 2.7 | | | 14 | | |
| Lo et al. (2010) § | | 1.11 | | 4.46 | 47 | | 1 | | 3.78 | | | 27 | | |
| **(additional effects of Robot Assisted Therapy vs other intervention)** | | | | | | | | | | | | | | |
| ***Short- term*** | |  | |  |  | |  | |  | | |  | | |
| Kahn et al. (2006) | | 0.2 | | 0.4 | 10 | | 0.3 | | 0.5 | | | 9 | | |
| Liao et al. (2011) | | 51.2 | | 8.82 | 10 | | 40.9 | | 13.14 | | | 10 | | |
| Masiero et al. (2014) ° | | 64 | | 12.41 | 14 | | 57 | | 24.49 | | | 16 | | |
| McCabe et al. (2015) | | 31.3 | | 6.2 | 12 | | 33.5 | | 8.3 | | | 11 | | |
| Page et al. (2012) § | | 22.86 | | 7.01 | 8 | | 21 | | 7.54 | | | 8 | | |
| Reinkensmeyer et al. (2012) | | 26.5 | | 11.2 | 13 | | 23 | | 8 | | | 13 | | |
| Timmermans et al. (2014)° | | 55 | | 8.46 | 11 | | 54 | | 6.77 | | | 11 | | |
| Yang et al. (2012) unilateral | | 47 | | 8.4 | 7 | | 46 | | 11.1 | | | 7 | | |
| Yang et al. (2012) bilateral | | 44.6 | | 10 | 7 | | 46 | | 11.1 | | | 7 | | |
| ***Medium- term*** | |  | |  |  | |  | |  | | |  | | |
| Masiero et al. (2014) | | 66 | | 8.59 | 14 | | 57 | | 9.18 | | | 16 | | |
| Timmermans et al. (2014) | | 52 | | 13.54 | 11 | | 53 | | 7.45 | | | 11 | | |

SD = standard deviation

*Comparison = minimal intervention or other intervention

§SD estimated based on standard errors

‡SD estimated based on confidence intervals

°SD estimated based on interquartile

þSD was imputed using similar sample from other included studies

£SD was imputed using p value

¢SD was imputed using p value of difference between groups

**Upper limb proximal**

| **Study** | **Exercise** | | | | | | | ***Comparison** | | | | |  |
| --- | --- | --- | --- | --- | --- | --- | --- | --- | --- | --- | --- | --- | --- |
|  | **Mean** | | **SD** | | | **Sample** | | **Mean** | | **SD** | **Sample** | |  |
| **(exercise vs minimal intervention)** | | | | | | | | | | | | |  |
| ***Short- term*** | | | | | | | | | | | | |  |
| Fasoli et al. (2004) § | | 14.4 | | 3.83 | 30 | | 11.6 | | 4.08 | | | 26 | |
| Ramos-Murguialday et al. (2013) | | 2.43 | | 3.28 | 16 | | 0.035 | | 3.25 | | | 14 | |
| Susanto et al. (2015) | | 3.44 | | 2.01 | 9 | | 3.3 | | 2.65 | | | 10 | |
| Volpe et al. (1999) § | | 9 | | 6.12 | 6 | | 5.8 | | 6.37 | | | 6 | |
| ***Medium- term*** | |  |  |  |  |  |  |  |  |  |  |  |  |
| Susanto et al. (2015) | | 3.67 | | 5.35 | 9 | | 1.4 | | 2.87 | | | 10 | |
| ***Long- term*** | |  | |  |  | |  | |  | | |  | |
| Volpe et al. (1999) § | | 12.2 | | 11.27 | 6 | | 12.5 | | 12.25 | | | 6 | |
| **(exercise vs other intervention)** | | | | | | | | | | | | |  |
| ***Short- term*** | | | | | | | | | | | | |  |
| Abdullah et al. (2011) § | 1.5 | | 0.93 | | | 8 | | 0.55 | | 0.93 | 11 | |  |
| Ang et al. (2014) | 4 | | 3.5 | | | 8 | | 2.6 | | 4.4 | 7 | |  |
| Burgar et al. (2000) | 4.5 | | 2.5 | | | 11 | | 1.3 | | 1.5 | 10 | |  |
| Burgar et al. (2011) high dose § | 9.1 | | 10.31 | | | 17 | | 8.8 | | 10.61 | 18 | |  |
| Burgar et al. (2011) low dose § | 4.2 | | 3.49 | | | 19 | | 8.8 | | 10.61 | 18 | |  |
| De Araújo et al. (2011) | 27 | | 3.29 | | | 6 | | 27.67 | | 7.09 | 6 | |  |
| Hesse et al. (2005) | 15.6 | | 6 | | | 21 | | 9.8 | | 5.9 | 22 | |  |
| Lin et al. (2015) | 27.44 | | 8.05 | | | 16 | | 24.41 | | 9.45 | 17 | |  |
| Lum et al. (2006) unilateral § | 4.3 | | 4.2 | | | 9 | | 2.5 | | 1.47 | 6 | |  |
| Lum et al. (2006) bilateral § | 2.4 | | 3.35 | | | 5 | | 2.5 | | 1.47 | 6 | |  |
| Volpe et al. (2008) § | 15.82 | | 6.96 | | | 11 | | 14.8 | | 5.06 | 10 | |  |
| Wu et al. (2012) | 33.07 | | 4.46 | | | 14 | | 33.14 | | 4.31 | 14 | |  |
| ***Medium- term*** |  | |  | | |  | |  | |  |  | |  |
| Ang et al. (2014) | 5.8 | | 2.9 | | | 8 | | 3.3 | | 4 | 7 | |  |
| Burgar et al. (2011) high dose § | 14.5 | | 11.94 | | | 11 | | 8.1 | | 11.43 | 12 | |  |
| Burgar et al. (2011) low dose § | 10.1 | | 7.48 | | | 14 | | 8.1 | | 11.43 | 12 | |  |
| Hesse et al. (2005) | 20.2 | | 8.6 | | | 19 | | 14.7 | | 9.2 | 20 | |  |
| Lum et al. (2006) unilateral § | 7.3 | | 5.29 | | | 7 | | 7.6 | | 2.68 | 5 | |  |
| Lum et al. (2006) bilateral § | 4.4 | | 2.91 | | | 5 | | 7.6 | | 2.68 | 5 | |  |
| **(additional effects of Robot Assisted Therapy vs other intervention)** | | | | | | | | | | | | |  |
| ***Short- term*** | | | | | | | | | | | | |  |
| Masiero et al. (2014) ° | 42 | | 8.59 | | | 14 | | 39 | | 18.37 | 16 | |  |
| McCabe et al. (2015) | 16.6 | | 2.5 | | | 12 | | 16.4 | | 3.9 | 11 | |  |
| Rabadi et al. (2008) § | 8.03 | | 5.57 | | | 10 | | 9.05 | | 6.04 | 10 | |  |
| Yang et al. (2012) unilateral | 33.9 | | 3.9 | | | 7 | | 32.4 | | 4 | 7 | |  |
| Yang et al. (2012) bilateral | 32.6 | | 4.8 | | | 7 | | 32.4 | | 4 | 7 | |  |
| ***Medium- term*** |  | |  | | |  | |  | |  |  | |  |
| Masiero et al. (2014) ° | 42 | | 5.73 | | | 14 | | 39 | | 18.37 | 16 | |  |

SD = standard deviation

*Comparison = minimal intervention or other intervention

§SD estimated based on standard errors

°SD estimated based on interquartile

£SD was imputed using p value

**Upper limb distal**

| **Study** | **Exercise** | | | | | | | | | ***Comparison** | | | | | | | | | |
| --- | --- | --- | --- | --- | --- | --- | --- | --- | --- | --- | --- | --- | --- | --- | --- | --- | --- | --- | --- |
|  | **Mean** | | **SD** | | | | **Sample** | | | **Mean** | | | **SD** | | | **Sample** | | | |
| **(exercise vs minimal intervention)** | | | | | | | | | | | | | | | | | | | |
| ***Short- term*** | | | | | | | | | | | | | | | | | | | |
| Fasoli et al. (2004) § | | 5.7 | | 5.48 | | 30 | | | 4.8 | | | 5.61 | | | | | | 26 | |
| Ramos-Murguialday et al. (2013) | | 0.73 | | 1.76 | | 16 | | | 0.39 | | | 1.16 | | | | | | | 14 |
| Susanto et al. (2015) | | 1.44 | | 4.14 | | 9 | | | 2 | | | 1.67 | | | | | | | 10 |
| Volpe et al. (1999) § | | 6.2 | | 3.92 | | 6 | | | 2.8 | | | 5.14 | | | | | | | 6 |
| ***Medium- term*** | | | | | | | | | | | | | | | | | | | |
| Susanto et al. (2015) | | 2.67 | 4.97 | | 9 | | | 0.8 | | | 1.99 | | | 10 | | | | | |
| ***Long- term*** | |  | |  |  | | | |  | | |  | | |  | | | | |
| Volpe et al. (1999) § | | 8 | | 9.06 | 6 | | | | 8 | | | 9.8 | | | 6 | | | | |
| **(exercise vs other intervention)** | | | | | | | | | | | | | | | | | | | |
| ***Short- term*** | | | | | | | | | | | | | | | | | | | |
| Abdullah et al. (2011) § | | 1.25 | 0.76 | | | | 8 | | | 0.45 | | | 0.76 | | | | 11 | | |
| Ang et al. (2014) | | 2.5 | 2.6 | | | | 8 | | | 1 | | | 1.3 | | | | 7 | | |
| Burgar et al. (2000) § | | 1 | 3 | | | | 11 | | | 1 | | | 2.8 | | | | 10 | | |
| De Araújo et al. (2011) | | 10.17 | 2.32 | | | | 6 | | | 17.33 | | | 6.59 | | | | 6 | | |
| Hesse et al. (2005) | | 9 | 4.8 | | | | 21 | | | 0.6 | | | 0.8 | | | | 22 | | |
| Lin et al. (2015) | | 19.31 | 8.52 | | | | 16 | | | 16 | | | 11.74 | | | | 17 | | |
| Lum et al. (2006) unilateral § | | 3.6 | 3.9 | | | | 9 | | | 3.3 | | | 4.65 | | | | 6 | | |
| Lum et al. (2006) bilateral § | | 1.4 | 1.57 | | | | 5 | | | 3.3 | | | 4.65 | | | | 6 | | |
| Sale et al. (2014) | | 71.64 | 28.81 | | | | 11 | | | 97.13 | | | 14.58 | | | | 9 | | |
| Volpe et al. (2008) § | | 3 | 5.97 | | | | 11 | | | 2.3 | | | 2.85 | | | | 10 | | |
| Wu et al. (2012) | | 14.07 | 7.66 | | | | 14 | | | 15.43 | | | 9.1 | | | | 14 | | |
| ***Medium- term*** | |  |  | | | |  | | |  | | |  | | | |  | | |
| Ang et al. (2014) | | 2.5 | 3 | | | | 8 | | | 0.3 | | | 2.1 | | | | 7 | | |
| Hesse et al. (2005) | | 9.8 | 6 | | | | 19 | | | 1.9 | | | 2.2 | | | | 20 | | |
| Lum et al. (2006) unilateral § | | 8.9 | 5.56 | | | | 7 | | | 6.2 | | | 5.59 | | | | 5 | | |
| Lum et al. (2006) bilateral § | | 3 | 3.35 | | | | 5 | | | 6.2 | | | 5.59 | | | | 5 | | |
| **(additional effects of Robot Assisted Therapy vs other intervention)** | | | | | | | | | | | | | | | | | | | |
| ***Short- term*** | | | | | | | | | | | | | | | | | | | |
| Masiero et al. (2014) ° | | 23 | 3.82 | | | | 14 | | | 18 | | | 9.18 | | | | 16 | | |
| McCabe et al. (2015) | | 12 | 4.1 | | | | 12 | | | 14.7 | | | 4.7 | | | | 11 | | |
| Rabadi et al. (2008) § | | 3.02 | 5.79 | | | | 10 | | | 3.89 | | | 6.48 | | | | 10 | | |
| Yang et al. (2012) unilateral | | 13.6 | 8.6 | | | | 7 | | | 12.9 | | | 7.7 | | | | 7 | | |
| Yang et al. (2012) bilateral | | 13.1 | 8.4 | | | | 7 | | | 12.9 | | | 7.7 | | | | 7 | | |
| ***Medium- term*** | |  |  | | | |  | | |  | | |  | | | |  | | |
| Masiero et al. (2014) ° | | 24 | 2.86 | | | | 14 | | | 18 | | | 8.16 | | | | 16 | | |

SD = standard deviation

*Comparison = minimal intervention or other intervention

§SD estimated based on standard errors

°SD estimated based on interquartile

**SPASTICITY (n = 14)**

**Upper limb total**

| **Study** | **Exercise** | | | | | | | ***Comparison** | | | |
| --- | --- | --- | --- | --- | --- | --- | --- | --- | --- | --- | --- |
|  | **Mean** | | **SD** | | | **Sample** | | **Mean** | | **SD** | **Sample** |
| **(exercise vs minimal intervention)** | | | | | | | | | | | |
| ***Short- term*** | | | | | | | | | | | |
| Ramos-Murguialday et al. (2013) | | 9.13 | | 7.32 | 16 | | 6.36 | | 5.54 | | 14 |
| **(exercise vs other intervention)** | | | | | | | | | | | |
| ***Short- term*** | | | | | | | | | | | |
| Burgar et al. (2011) high dose § | 0.19 | | 0.37 | | | 17 | | 0.11 | | 0.42 | 18 |
| Burgar et al. (2011) low dose § | 0 | | 0.26 | | | 19 | | 0.11 | | 0.42 | 18 |
| Byl et al. (2013) unilateral | 5 | | 14.1 | | | 5 | | 6.2 | | 17.5 | 5 |
| Byl et al. (2013) bilateral | 7 | | 19.8 | | | 5 | | 6.2 | | 17.5 | 5 |
| Hesse et al. (2005) | 1.7 | | 2.4 | | | 21 | | 1.8 | | 1.7 | 22 |
| Klamroth-Marganska et al. (2014) ‡ | -0.62 | | 0.16 | | | 38 | | 0 | | 0.16 | 35 |
| Lo et al. (2010) | -0.03 | | 0.55 | | | 25 | | -0.04 | | 0.57 | 27 |
| Volpe et al. (2008) § | 6.27 | | 3.32 | | | 11 | | 6 | | 4.11 | 10 |
| ***Medium- term*** |  | |  | | |  | |  | |  |  |
| Burgar et al. (2011) high dose § | 0.83 | | 0.83 | | | 11 | | 0.16 | | 0.52 | 12 |
| Burgar et al. (2011) low dose § | 0.02 | | 0.52 | | | 14 | | 0.16 | | 0.52 | 12 |
| Hesse et al. (2005) | 1.4 | | 2.6 | | | 19 | | 1.8 | | 1.7 | 20 |
| **(additional effects of Robot Assisted Therapy vs other intervention)** | | | | | | | | | | | |
| ***Short- term*** | | | | | | | | | | | |
| Masiero et al. (2014) ‡ | 0 | | 0.1 | | | 14 | | 1 | | 1.02 | 16 |
| Rabadi et al. (2008) § | 2.73 | | 4.08 | | | 10 | | 3.18 | | 4.43 | 10 |
| Yang et al. (2012) unilateral | 0.5 | | 0.3 | | | 7 | | 0.5 | | 0.4 | 7 |
| Yang et al. (2012) bilateral | 0.5 | | 0.2 | | | 7 | | 0.5 | | 0.4 | 7 |
| ***Medium- term*** |  | |  | | |  | |  | |  |  |
| Masiero et al. (2014) ‡ | 0 | | 0.1 | | | 14 | | 0.5 | | 1.02 | 16 |

SD = standard deviation

*Comparison = minimal intervention or other intervention

§SD estimated based on standard errors

‡SD estimated based on confidence intervals

**Upper limb proximal**

| **Study** | **Exercise** | | | ***Comparison** | | |
| --- | --- | --- | --- | --- | --- | --- |
|  | **Mean** | **SD** | **Sample** | **Mean** | **SD** | **Sample** |
| **(exercise vs other intervention)** | | | | | | |
| ***Short- term*** | | | | | | |
| De Araújo et al. (2011) | 2 | 0.1 | 6 | 1.5 | 0.55 | 6 |
| Hesse et al. (2005) | 0.9 | 1.3 | 21 | 0.6 | 0.7 | 22 |
| Lum et al. (2006) unilateral § | 0.9 | 1.8 | 9 | -1.3 | 1.71 | 6 |
| Lum et al. (2006) bilateral § | -0.4 | 0.89 | 5 | -1.3 | 1.71 | 6 |
| Sale et al. (2014) | 0.73 | 0.96 | 26 | 0.93 | 0.96 | 27 |
| ***Medium- term*** |  |  |  |  |  |  |
| Hesse et al. (2005) | 0.8 | 1.4 | 19 | 0.5 | 0.6 | 20 |
| Lum et al. (2006) unilateral § | 0.3 | 2.91 | 7 | 0.2 | 1.79 | 5 |
| Lum et al. (2006) bilateral § | -2 | 1.79 | 5 | 0.2 | 1.79 | 5 |

SD = standard deviation

*Comparison = minimal intervention or other intervention

§SD estimated based on standard errors

**Upper limb distal**

| **Study** | | **Exercise** | | | ***Comparison** | | | | |  |
| --- | --- | --- | --- | --- | --- | --- | --- | --- | --- | --- |
|  | | **Mean** | **SD** | **Sample** | | **Mean** | **SD** | **Sample** | |  |
| **(exercise vs other intervention)** | | | | | | | | | | |
| ***Short- term*** | | | | | | | | | | |
| De Araújo et al. (2011) | 2.33 | | 0.52 | 6 | | 1.5 | 0.55 | | 6 | |
| Hesse et al. (2005) | 0.8 | | 1.4 | 21 | | 1.2 | 1.3 | | 22 | |
| Lum et al. (2006) unilateral § | 0 | | 2.4 | 9 | | 0.7 | 1.47 | | 6 | |
| Lum et al. (2006) bilateral § | -1 | | 1.34 | 5 | | 0.7 | 1.47 | | 6 | |
| Sale et al. (2014) | 5.63 | | 4.61 | 11 | | 3.62 | 3.33 | | 9 | |
| ***Medium- term*** |  | |  |  | |  |  | |  | |
| Hesse et al. (2005) | 0.6 | | 1.4 | 19 | | 1.3 | 1.4 | | 20 | |
| Lum et al. (2006) unilateral § | -0.6 | | 1.59 | 7 | | 0.8 | 1.57 | | 5 | |
| Lum et al. (2006) bilateral § | -1.2 | | 1.79 | 5 | | 0.8 | 1.57 | | 5 | |

SD = standard deviation

*Comparison = minimal intervention or other intervention

§SD estimated based on standard errors

| **STRENGTH (n = 20)**  **Upper limb total** | | | | | | |
| --- | --- | --- | --- | --- | --- | --- |
| **Study** | **Exercise** | | | ***Comparison** | | |
|  | **Mean** | **SD** | **Sample** | **Mean** | **SD** | **Sample** |
| **(exercise vs other intervention)** | | | | | | |
| ***Short- term*** | | | | | | |
| Burgar et al. (2011) high dose § | 16 | 12.37 | 17 | 15.4 | 15.7 | 18 |
| Burgar et al. (2011) low dose § | 13.7 | 10.03 | 19 | 15.4 | 15.7 | 18 |
| Byl et al. (2013) unilateral | 148.1 | 53.32 | 5 | 104.3 | 47.06 | 5 |
| Byl et al. (2013) bilateral | 143 | 43.8 | 5 | 104.3 | 47.06 | 5 |
| Hesse et al. (2005) | 21.8 | 10.5 | 21 | 6.8 | 8.3 | 22 |
| Hsieh et al. (2011) high intensity | 3.81 | 0.55 | 6 | 3.33 | 0.69 | 6 |
| Hsieh et al. (2011) lower intensity | 3.25 | 0.5 | 6 | 3.33 | 0.69 | 6 |
| Lum et al. (2006) unilateral § | 10.1 | 7.2 | 9 | 9.3 | 3.18 | 6 |
| Lum et al. (2006) bilateral§ | 3.2 | 2.24 | 5 | 9.3 | 3.18 | 6 |
| Xu et al. (2012) | 7.59 | 0.55 | 9 | 5.64 | 0.46 | 9 |
| ***Medium- term*** |  |  |  |  |  |  |
| Burgar et al. (2011) high dose § | 27.8 | 13.27 | 11 | 24.4 | 16.63 | 12 |
| Burgar et al. (2011) low dose § | 18 | 12.35 | 14 | 24.4 | 16.63 | 12 |
| Hesse et al. (2005) | 22.6 | 11.1 | 19 | 7.9 | 9 | 20 |
| Lum et al. (2006) unilateral § | 17.9 | 9 | 7 | 14.2 | 5.14 | 5 |
| Lum et al. (2006) bilateral § | 11.2 | 7.16 | 5 | 14.2 | 5.14 | 5 |
| **(additional effects of Robot Assisted Therapy vs other intervention)** | | | | | | |
| ***Short- term*** | | | | | | |
| Yang et al. (2012) unilateral | 3.6 | 0.6 | 7 | 3.7 | 0.9 | 7 |
| Yang et al. (2012) bilateral | 3.7 | 0.6 | 7 | 3.7 | 0.9 | 7 |

SD = standard deviation

*Comparison = minimal intervention or other intervention

§SD estimated based on standard errors

**Upper limb proximal**

| **Study** | | **Exercise** | | | | | | | | | | ***Comparison** | | | | | | | | | |  |  |
| --- | --- | --- | --- | --- | --- | --- | --- | --- | --- | --- | --- | --- | --- | --- | --- | --- | --- | --- | --- | --- | --- | --- | --- |
|  | | **Mean** | | | **SD** | | | **Sample** | | | | **Mean** | | | | | **SD** | | | | **Sample** |  |  |
| **(exercise vs mininal intervention)** | | | | | | | | | | | | | | | | | | | | | | |  |
| ***Short- term*** | | | | | | | | | | | | | | | | | | | | | | |  |
| Aisen et al. (1997) | | | 3.88 | 2.89 | | | | | | 10 | | | | 2.3 | 2.45 | | | | | 10 | | |  |
| Fasoli et al. (2004) ° | | | 6.8 | 2.19 | | | | | | 30 | | | | 5.1 | 2.55 | | | | | 26 | | |  |
| Volpe et al. (1999) § | | | 9.1 | 4.9 | | | | | | 6 | | | | 5.1 | 3.92 | | | | | 6 | | |  |
| **(exercise vs other intervention)** | | | | | | | | | | | | | | | | | | | | | | |  |
| ***Short- term*** | | | | | | | | | | | | | | | | | | | | | | | |
| Byl et al. (2013) unilateral þ | 8 | | | | | 1 | | | 5 | | | | 18 | | | 0.5 | | | 5 | | | | |
| Byl et al. (2013) bilateral þ | 10 | | | | | 0.2 | | | 5 | | | | 18 | | | 0.5 | | | 5 | | | | |
| Hesse et al. (2005) | 12.1 | | | | | 3.9 | | | 21 | | | | 4.4 | | | 2.2 | | | 22 | | | | |
| Klamroth-Marganska et al. (2014) | 0.31 | | | | | 1.73 | | | 38 | | | | 0.58 | | | 2.15 | | | 35 | | | | |
| Sale et al. (2014) | 57.77 | | | | | 24.22 | | | 26 | | | | 39.56 | | | 35.1 | | | 27 | | | | |
| Simkins et al. (2013) unilateral þ | 20 | | | | | 1 | | | 5 | | | | 55 | | | 0.5 | | | 5 | | | | |
| Simkins et al. (2013) bilateral þ | 5 | | | | | 0.2 | | | 5 | | | | 55 | | | 0.5 | | | 5 | | | | |
| Volpe et al. (2008) § | 35.45 | | | | | 13.6 | | | 11 | | | | 33.7 | | | 8.54 | | | 10 | | | | |
| Xu et al. (2014) § | 5.65 | | | | | 1.2 | | | 23 | | | | 4.54 | | | 0.98 | | | 22 | | | | |
| ***Medium- term*** |  | | |  | | |  | | | |  | | | |  | | |  | | | | | |
| Hesse et al. (2005) | 12.7 | | | 5.3 | | | 19 | | | | 5.1 | | | | 4.1 | | | 20 | | | | | |
| Klamroth-Marganska et al. (2014) | 0.44 | | | 1.7 | | | 38 | | | | 0.85 | | | | 2.67 | | | 35 | | | | | |
| **(additional effects of Robot Assisted Therapy vs other intervention)** | | | | | | | | | | | | | | | | | | | | | | | |
| ***Short- term*** | | | | | | | | | | | | | | | | | | | | | | | |
| Masiero et al. (2014) § | 5 | | | 0.95 | | | 14 | | | | 4 | | | | 1.02 | | | 16 | | | | | |
| Rabadi et al. (2008) | 18.93 | | | 11.76 | | | 10 | | | | 15.74 | | | | 12.74 | | | 10 | | | | | |
| Yang et al. (2012) unilateral | 4.1 | | | 1 | | | 7 | | | | 4.6 | | | | 0.5 | | | 7 | | | | | |
| Yang et al. (2012) bilateral | 4.8 | | | 0.2 | | | 7 | | | | 4.6 | | | | 0.5 | | | 7 | | | | | |
| ***Medium- term*** |  | | |  | | |  | | | |  | | | |  | | |  | | | | | |
| Masiero et al. (2014) § | 5 | | | 0.95 | | | 14 | | | | 5 | | | | 1.02 | | | 16 | | | | | |

SD = standard deviation

*Comparison = minimal intervention or other intervention

§SD estimated based on standard errors

°SD estimated based on interquartile

þSD was imputed using similar sample from other included studies

**Upper limb distal**

| **Study** | **Exercise** | | | | | | ***Comparison** | | | | | |  |
| --- | --- | --- | --- | --- | --- | --- | --- | --- | --- | --- | --- | --- | --- |
|  | **Mean** | | **SD** | | **Sample** | | **Mean** | | | **SD** | | **Sample** |  |
| **(exercise vs other intervention)** | | | | | | | | | | | | | |
| ***Short- term*** | | | | | | | | | | | | | |
| Byl et al. (2013) unilateral þ | | -1 | | 1.2 | | 5 | | 2 | 1.5 | | 5 | | |
| Byl et al. (2013) bilateral þ | | 8 | | 1.1 | | 5 | | 2 | 1.5 | | 5 | | |
| Hesse et al. (2005) | | 9.7 | | 5.3 | | 21 | | 2.4 | 2.2 | | 22 | | |
| Housman et al. (2009) | | 1.76 | | 6.61 | | 15 | | 1.76 | 5.07 | | 16 | | |
| Klamroth-Marganska et al. (2014) | | -0.41 | | 1.39 | | 38 | | 0 | 1.39 | | 35 | | |
| Sale et al. (2014) | | 6.9 | | 2.77 | | 11 | | 7.87 | 2.58 | | 9 | | |
| Simkins et al. (2013) unilateral þ | | -20 | | 1.2 | | 5 | | 10 | 1.5 | | 5 | | |
| Simkins et al. (2013) bilateral þ | | 30 | | 1.1 | | 5 | | 10 | 1.5 | | 5 | | |
| ***Medium- term*** | |  | |  | |  | |  |  | |  | | |
| Hesse et al. (2005) | | 9.9 | | 5.9 | | 19 | | 2.8 | 3.9 | | 20 | | |
| Housman et al. (2009) | | 3.96 | | 10.58 | | 14 | | 3.08 | 4.85 | | 14 | | |
| **(additional effects of Robot Assisted Therapy vs other intervention)** | | | | | | | | | | | | | |
| ***Short- term*** | | | | | | | | | | | | | |
| Masiero et al. (2014) ° | | 5 | | 0.95 | | 14 | | 4 | 1.02 | | 16 | | |
| Reinkensmeyer et al. (2012) | | 7.05 | | 6.61 | | 13 | | 10.36 | 10.36 | | 13 | | |
| Yang et al. (2012) unilateral | | 3.2 | | 1.2 | | 7 | | 2.8 | 1.5 | | 7 | | |
| Yang et al. (2012) bilateral | | 2.6 | | 1.1 | | 7 | | 2.8 | 1.5 | | 7 | | |
| Yoo et al. (2013) | | 18.73 | | 12.78 | | 11 | | 11.24 | 5.07 | | 11 | | |
| ***Medium- term*** | |  | |  | |  | |  |  | |  | | |
| Masiero et al. (2014) ° | | 5 | | 0.95 | | 14 | | 5 | 1.02 | | 16 | | |

SD = standard deviation

*Comparison = minimal intervention or other intervention

°SD estimated based on interquartile

þSD was imputed using similar sample from other included studies

**RANGE OF MOTION (n = 5)**

**Upper limb total**

| **Study** | **Exercise** | | | | | | ***Comparison** | | | | | | |  |
| --- | --- | --- | --- | --- | --- | --- | --- | --- | --- | --- | --- | --- | --- | --- |
|  | **Mean** | | **SD** | | **Sample** | | | **Mean** | | **SD** | | **Sample** | |  |
| **(exercise vs other intervention)** | | | | | | | | | | | | | | |
| ***Short- term*** | | | | | | | | | | | | | | |
| Byl et al. (2013) unilateral | | 733.0 | | 65.54 | | 5 | | | 750.8 | | 129.37 | | 5 | |
| Byl et al. (2013) bilateral | | 703.2 | | 87.66 | | 5 | | | 750.8 | | 129.37 | | 5 | |
| Housman et al. (2009) | | -2.8 | | 3.4 | | 15 | | | -1.6 | | 2.8 | | 16 | |
| Sale et al. (2014) | | 809.04 | | 90.5 | | 26 | | | 792.59 | | 83.0 | | 27 | |

SD = standard deviation

*Comparison = minimal intervention or other intervention

**Upper limb proximal (elbow flexion)**

| **Study** | | **Exercise** | | | | | | ***Comparison** | | | | |  |
| --- | --- | --- | --- | --- | --- | --- | --- | --- | --- | --- | --- | --- | --- |
|  | | **Mean** | | **SD** | | **Sample** | | **Mean** | | **SD** | | **Sample** |  |
| **(exercise vs other intervention)** | | | | | | | | | | | | | |
| Byl et al. (2013) unilateral þ | 0 | | 3.55 | | 5 | | 20 | | 2.67 | | 5 | | |
| Byl et al. (2013) bilateral þ | 17 | | 3.55 | | 5 | | 20 | | 2.67 | | 5 | | |
| Simkins et al. (2013) unilateral þ | 0 | | 3.55 | | 5 | | 5 | | 2.67 | | 5 | | |
| Simkins et al. (2013) bilateral þ | 0 | | 3.55 | | 5 | | 5 | | 2.67 | | 5 | | |
| Xu et al. (2014) § | 59.01 | | 3.55 | | 23 | | 52.71 | | 2.67 | | 22 | | |

SD = standard deviation

*Comparison = minimal intervention or other intervention

§SD estimated based on standard errors

þSD was imputed using similar sample from other included studies

**Upper limb distal (wrist flexion)**

| **Study** | | **Exercise** | | | | | | ***Comparison** | | | | |
| --- | --- | --- | --- | --- | --- | --- | --- | --- | --- | --- | --- | --- |
|  | | **Mean** | | **SD** | | **Sample** | | **Mean** | | **SD** | | **Sample** |
| **(exercise vs other intervention)** | | | | | | | | | | | | |
| Byl et al (2013) unilateral þ | 10 | | 3.55 | | 5 | | 18 | | 2.67 | | 5 | |
| Byl et al (2013) bilateral þ | -5 | | 3.55 | | 5 | | 18 | | 2.67 | | 5 | |
| Simkins et al. (2013) unilateral þ | 0 | | 3.55 | | 5 | | 60 | | 2.67 | | 5 | |
| Simkins et al. (2013) bilateral þ | 0 | | 3.55 | | 5 | | 60 | | 2.67 | | 5 | |

SD = standard deviation

*Comparison = minimal intervention or other intervention

þSD was imputed using similar sample from other included studies

| **PAIN (n = 6)** |  | | |  | | |
| --- | --- | --- | --- | --- | --- | --- |
| **Study** | **Exercise** | | | ***Comparison** | | |
|  | **Mean** | **SD** | **Sample** | **Mean** | **SD** | **Sample** |
| **(exercise vs other intervention)** | | | | | | |
| ***Short- term*** | | | | | | |
| Abdullah et al. (2011) # | 0.5 | 0.85 | 8 | 0.27 | 0.86 | 11 |
| Byl et al. (2013) unilateral | 2.8 | 2.0 | 5 | 2.6 | 1.82 | 5 |
| Byl et al. (2013) bilateral | 1.4 | 1.14 | 5 | 2.6 | 1.82 | 5 |
| Lo et al. (2010) | -0.81 | 1.95 | 25 | 0 | 1.97 | 27 |
| Simkins et al. (2013) unilateral þ | 0 | 1.82 | 5 | 0 | 2 | 5 |
| Simkins et al. (2013) bilateral þ | 0 | 1.14 | 5 | 0 | 2 | 5 |
| Volpe et al. (2008) § | 23.1 | 1.33 | 11 | 22.8 | 1.58 | 10 |
| **(additional effects of Robot Assisted Therapy vs other intervention)** | | | | | | |
| ***Short- term*** | | | | | | |
| Rabadi et al. (2008) § | 22.98 | 4.65 | 10 | 21.93 | 4.65 | 10 |

SD = standard deviation

*Comparison = minimal intervention or other intervention

§SD estimated based on standard errors

#SD estimated based on standard errors change

ÞSD was imputed using similar sample from other included studies
